# Associating transcription factors and conserved RNA structures with gene regulation in the human brain

**DOI:** 10.1038/s41598-017-06200-4

**Published:** 2017-07-18

**Authors:** Nikolai Hecker, Stefan E. Seemann, Asli Silahtaroglu, Walter L. Ruzzo, Jan Gorodkin

**Affiliations:** 1 Center for non-coding RNA in Technology and Health, University of Copenhagen, Grønnegårdsvej 3, 1870 Frederiksberg C, Denmark; 2 Department of Veterinary and Animal Sciences, University of Copenhagen, Grønnegårdsvej 3, 1870 Frederiksberg C, Denmark; 3 Max-Planck Institute of Molecular Cell Biology and Genetics, Pfotenhauerstr. 108, 01307 Dresden, Germany; 4 Department of Cellular and Molecular Medicine, University of Copenhagen, Blegdamsvej 3B, 2200 Copenhagen N, Denmark; 5 Paul G. Allen School of Computer Science & Engineering, and Department of Genome Sciences, University of Washington, 185 Stevens Way, WA 98195-2350 Seattle, USA; 6 Fred Hutchinson Cancer Research Center, 1100 Fairview Ave. N., WA 98109 Seattle, USA; 7 Max-Planck Institute for the Physics of Complex Systems, Nöthnitzer Str. 38, 01187 Dresden, Germany

## Abstract

Anatomical subdivisions of the human brain can be associated with different neuronal functions. This functional diversification is reflected by differences in gene expression. By analyzing post-mortem gene expression data from the Allen Brain Atlas, we investigated the impact of transcription factors (TF) and RNA secondary structures on the regulation of gene expression in the human brain. First, we modeled the expression of a gene as a linear combination of the expression of TFs. We devised an approach to select robust TF-gene interactions and to determine localized contributions to gene expression of TFs. Among the TFs with the most localized contributions, we identified EZH2 in the cerebellum, NR3C1 in the cerebral cortex and SRF in the basal forebrain. Our results suggest that EZH2 is involved in regulating ZIC2 and SHANK1 which have been linked to neurological diseases such as autism spectrum disorder. Second, we associated enriched regulatory elements inside differentially expressed mRNAs with RNA secondary structure motifs. We found a group of purine-uracil repeat RNA secondary structure motifs plus other motifs in neuron related genes such as ACSL4 and ERLIN2.

## Introduction

Transcription factors (TF) and regulatory elements (RE) located in mRNAs are among the most fundamental features for governing the expression of genes, and thereby the function and fate of a cell. Transcription factors and different co-factors often form large protein-complexes that regulate the transcription^1^. Post-transcriptional regulation is achieved by a similar combinatorial complexity of RNA binding proteins (RBP) and protein-protein interactions^2^. Common principles include stabilizing mRNAs or enhancing their degradation, as well as modifying transcripts, e.g., by splicing or alternative poly adenylation (APA)^3^. With the advent of high-throughput cross-linking and immuno-precipitation sequencing (CLIP-seq) and RNA immuno-precipitation sequencing (RIP-seq) technologies, experiments have been performed with the aim to characterize RBP complexes and their RNA targets including more than 1000 RBPs^4^.

The human brain is a prime candidate for studying both transcriptional and post-transcriptional regulation due to its complex functional sub-divisions^5^. Although many processes in the brain rely on an interplay between interconnected processing modules, some areas appear to be of special importance for certain functions inside the central nervous system (CNS). For example, the cerebellum was traditionally associated with motor control whereas the parietal and frontal lobe inside the cerebral cortex are often associated with more abstract processes such as short-term and long-term memory^6–8^. For some brain regions, there is an apparent difference in the cyto-architecture. For instance, the ratio between non-neuron cells and neuron cells is ≈3.76 inside the human cerebral cortex compared with ≈0.23 inside cerebellum^9^. The function and differentiation of a cell such as a neuron is dictated by the regulation of gene expression. Hence, studying gene expression inside the brain has been recognized as an important part of neuroscience. The Allen Institute for Brain Science created an extensive resource of gene expression data sets that cover a wide-range of human and mouse brain regions^10^. Hawrylycz *et al*. identified gene expression modules that correspond to distinct cell types and assessed overall differences in gene expression inside the human brain^11^. However, there has not been a systematic analysis that addresses the underlying features that govern gene expression inside the different regions of the human brain. Therefore, we analyzed micro array data from the Allen Brain Atlas^10^ with the aim to identify transcription factors and regulatory elements inside mRNAs that are important for gene expression in the brain, especially ones characteristic for specific brain regions.

In line with existing studies that aim to predict transcription factor (TF) interactions, we analyzed Chromatin immuno-precipitation sequencing (ChIP-seq) and gene expression data using a regression approach^12, 13^. In this study, we used ENCODE ChIP-seq data from the UCSC Genome Browser^14–17^. A more recent study suggested that combining gene expression data from TF perturbation experiments, e.g., over-expression or knock-down of TFs, with LASSO regression exhibits more accurate results than approaches based on TF binding affinities^18^. However, generating such TF perturbation gene expression data requires performing extensive experiments that are limited to cell lines and model organisms. Hence, it is not applicable to the large brain region post-mortem data set analyzed in our study. On the other hand, the ENCODE ChIP-seq data, used in this study, is not specific to the different brain regions. Therefore, we cannot infer read counts directly; instead, we focus on the most robust TF-gene interactions. For this purpose, we select the best-scoring ENCODE ChIP-seq peak cluster to infer potential TF-gene interactions and apply a more rigorous treatment of regression models. Our approach uses LASSO regression to select the most relevant set of interactions for each TF^19^ and then compares linear models derived from the set of selected interactions to random models. Brain region specific TF activity is one of the major mechanisms that regulates differences in gene expression, thus functional diversity in the brain. We devised an approach to assess the localization of the contribution to gene expression of a specific TF given different brain regions. In this context, we derive the contribution of a TF to gene expression from the regression coefficients, thereby quantifying the impact of a TF rather than its expression. This allows one to identify the TFs with the highest impact in a region, and to identify TFs with localized contributions to specific regions. We use Shannon entropy for identifying region-specific TF contributions, i.e., localized contributions, as previous studies have done to analyze region specific gene expression^20^.

Regulatory elements within mRNAs are another fundamental feature of gene regulation. Although there are some regulatory elements in mRNAs that form RNA structures, e.g., the roquin constitutive decay element^21^, regulatory RNA structures are less well characterized in general. In a previous study, our predictions showed that expressed protein-coding genes in mouse brains are enriched for the presence of conserved RNA secondary structures in their UTRs^22^. Therefore, here, we also aim to identify novel RNA secondary structure motifs that are potentially relevant for post-transcriptional regulation of transcripts in the human brain. Several computational approaches and studies have aimed to link gene expression data, RBP binding or decay rates with RNA secondary structure predictions^23–26^. An elaborate systematic study of sequence or structure based binding affinities of RBPs was performed on 30 mer RNA sequences that reflect eukaryotic transcriptomes^27^. A limitation of these existing approaches is, however, the lack of evolutionary evidence for the predicted RNA secondary structures. Comparative approaches for RNA secondary structure prediction have a lower false discovery rate than single-sequence based methods^28, 29^. In this study, we put an emphasis on cis-regulatory elements (RE) that are annotated in the AURA database^30^ and overlap with predicted conserved RNA secondary structures. For example, these REs include RNA protein binding sites and AU-rich elements. We refer to REs that overlap RNA secondary structure predictions as structured REs. The inferred structure predictions originate from a genome-wide computational screen for conserved RNA structures in vertebrate genome-alignments^31^.

## Results

Throughout this manuscript, we consider six different levels of coarse-graining for separating brain regions following the ontology as specified by the Allen Human Brain Atlas (Supplementary file S1, Fig. S1)^10^. We refer to these different levels of coarse-graining as depths where depth 1 refers to the entire gray matter of the brain. At depth 2, we are analyzing the telencephalon (Tel) also known as the cerebrum, diencephalon (DiE), metencephalon (MET), mesencephalon (MES) and myencephalon (MY). Depth 3 contains sub-regions of these five regions, e.g., the cerebral Cortex (Cx) and cerebral Nuclei (CxN) as sub-regions of the telencephalon. In the following section, we limited the results to robust findings, specifically, findings that could be reproduced in five independent instances of the analysis. Each instance of the analysis was initialized with different seeds for the random number generator, thus using different training or test sets during randomization steps.

### Transcription factor contribution to gene expression

**Figure 1:**
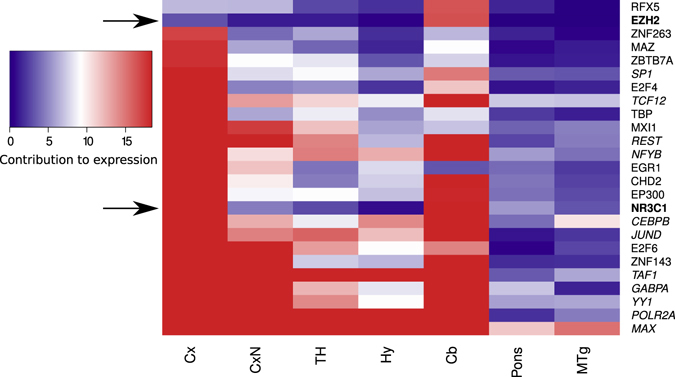
Transcription factor contributions to gene expression. The color gradient indicates the contribution to gene expression and is capped between 5% and 95% quantiles for all transcription factors (rows) in all depicted region (columns). Cx refers cerebral cortex, CxN to cerebral nuclei, TH to thalamus, Hy to hypothalamus, Cb to cerebellum and MTg to mid-brain tegmentum. Depicted are the contributions of the 25 transcription factors with the strongest contribution in any of the regions at depth 3 from one instance out of a five-times repeated analysis. Arrows point to transcription factors with localized contributions (bold font). Italic font indicates transcription factor with comparably strong contributions to gene expression in all regions and all instances of the analysis including different region hierarchy depths.

Based on a regression approach (see Material and Methods), we predicted eleven TFs with robust strong contributions to gene expression throughout all analyzed regions of depth 2, 3 and 4 (Fig. 1, Table 1) and three TFs with strong localized contributions to specific brain regions. This was done by modeling the expression of a gene as a linear combination of the expression of transcription factors. Since we used LASSO regression, the set of TFs is reduced to TFs that show a relevant contribution to the expression of the gene. The resulting coefficients of the linear models are then used to estimate the contribution of each transcription factor to the expression of the gene. To increase the robustness of our results, we compared the resulting linear models to random models and only considered models that performed better than the random models (Supplementary text S1, Fig. S3). We refer to the corresponding genes as TF regulated genes. Given the linear models, a gene is usually predicted to be regulated by more than one TF. For simplicity, we write that a TF regulates a gene if it is robustly predicted to be involved in the regulation of the gene. The contribution to gene expression of a TF inside a brain region is calculated as the sum over the contributions to the expression of all TF regulated genes. From the linear models, we also extracted the genes that were predicted to be regulated by a specific TF (Supplementary file S4) and computed the enrichment for GO-term using the ConsensusPathDB^32^ as described below.

**Table 1:** Transcription factor contributions to gene expression and the number of regulated genes.

TF	${\bar{{\boldsymbol{r}}}}_{{\boldsymbol{\max }}}$	Tel	MET	Cx	Cb	BF	T_*sel*_	${{\boldsymbol{n}}}_{{\boldsymbol{robust}}{\boldsymbol{:}}{{\boldsymbol{T}}}_{{\boldsymbol{sel}}}}$
MAX	2	149.86	48.49	110.93	61.87	31.22	Tel	180
TAF1	5	64.96	30.47	45.91	42.36	10.57	Tel	101
YY1	9	59.05	23.72	59.57	19.39	13.65	Tel	90
POLR2A	11	88.05	23.94	76.97	52.09	12.82	Tel	252
CEBPB	12	30.99	86.87	25.25	36.43	12.23	MET	64
JUND	14	35.75	17.39	41.50	21.93	13.47	Tel	34
NFYB	14	30.53	12.19	24.15	21.35	16.73	Tel	52
REST	14	38.72	47.50	21.51	25.24	9.5	MET	39
GABPA	15	49.29	26.25	41.12	52.55	18.06	Tel	101
TCF12	17	24.99	20.16	19.65	18.06	3.33	Tel	25
SP1	18	27.89	11.52	17.05	15.68	6.77	Tel	54
EZH2	81	3.42	19.02	2.83	19.62	1.47	MET	11
NR3C1	40	32.39	26.20	33.00	22.60	3.55	Cx	20
SRF	95	2.24	0.88	0.94	1.19	10.57	BF	5

Transcription factors (TF) with the strongest contribution to gene expression (ranked ≥20 in all regions) are given in the upper part and TFs with localized contributions are shown in the lower part of the table. ${\bar{r}}_{max}$ is the maximum (worst) rank in any region for the TF. Tel (telencephalon), MET (metencephalon), Cx (cerebral cortex), Cb (cerebellum) and BF (basal forebrain) show the contribution to gene expression of the TF in the specified region. These values are averages over five runs of the analysis initialized with different seed for the random number generator. T_*sel*_ indicates the region with the highest contribution to gene expression at depth 2 for brain-wide active TFs and a selected region with localized contributions for locally active TFS. ${n}_{robust:{T}_{sel}}$ refers to the number of TF-gene interactions that were consistently predicted in these five runs for the correspoding region.

#### Brain-wide most active transcription factors

First, we aimed to characterize the most active TFs throughout the brain, i.e., the TFs that were predicted to show the highest contributions in all analyzed brain regions. Eleven TFs ranked among the top 20 out 141 TFs regarding the highest contribution to gene expression in each region of depth 2, 3 and 4 (Supplementary text S1, Figs S4–S18). Although the eleven TFs exhibit strong contributions to gene expression in all analyzed regions, the strongest contributions were in general either predicted for the telencephalon or metencephalon (Table 1, Supplementary file S3). The TF MYC associated factor X (MAX) showed the strongest contribution to gene expression overall in the analyzed brain regions. MAX regulates 180 genes inside the telencephalon. These 180 genes are enriched for the GO-terms primary metabolic process (GO:0034660)(q-value < 0.05), as well as nucleoplasm, intracellular organelle part, nucleus, heterocyclic compound binding, organic cyclic compound binding, transferase activity and nucleic acid binding chromatin binding (q-value < 0.005) (Supplementary file S5). Other globally active TFs regulate genes that are enriched for similar GO-terms, i.e., the TF TATA-box binding protein associated factor 1 (TAF1), YY1 transcription factor (YY1), RNA polymerase II subunit A (POLR2A), and GA binding protein transcription factor alpha subunit (GABPA) (Supplementary file S5).

Also predicted to be among the globally most active TFs, CCAAT/enhancer binding protein beta (CEBPB), RE1 silencing transcription factor (REST) and Sp1 transcription factor (SP1) regulate more exclusively genes with neuronal function. CEBPB regulates the microtubule associated genes (GO:0097427) microtubule affinity regulating kinase 2 (MARK2) and microtubule cross-linking factor 1 (MTCL1 also known as SOGA2), and three steroid hormone receptor activity related genes (GO:0003707), i.e., nuclear receptor subfamily 2 group F member 6 (NR2F6), retinoic acid receptor beta (RARB) and nuclear receptor subfamily 1 group D member 2 (NR1D2) inside the telencephalon. A functional circuit involved in intraciliary transport (GO:0030990) comprises the proteins TULP3, IFT20 and IFT27, and is predicted to be commonly regulated by the TF SP1 among other genes.

REST is predicted to regulate 59 genes inside the telencephalon. The genes predicted to be regulated by REST are enriched for neuron related GO-terms (q-value < 0.05) of which 14 of the 59 genes are members, e.g., somatodendritic compartment (GO:0036477), neuron projection (GO:0043005) and calcium ion binding (GO:0005509) (Supplementary file S5). These 14 neuron related genes are: bone morphogenetic protein receptor type 1B (BMPR1B), C2 calcium dependent domain containing 4A (C2CD4A), cyclin dependent kinase 5 regulatory subunit 1 (CDK5R1), contactin associated protein 1 (CNTNAP1), G protein subunit alpha o1 (GNAO1), glutamate ionotropic receptor NMDA type subunit 1 (GRIN1), potassium voltage-gated channel subfamily C member 1 (KCNC1), NSF attachment protein alpha (NAPA), neurocan (NCAN), RNA polymerase II subunit M (POLR2M), protein kinase C substrate 80K-H (PRKCSH), solute carrier family 12 member 5 (SLC12A5), sushi, nidogen and EGF like domains 1 (SNED1) and tumor protein D52 (TPD52). Although not significantly enriched (q-value = 0.06), four REST regulated genes include members of the BDNF pathway, i.e., mitogen-activated protein kinase 8 (MAPK8), GRIN1, CDK5R1 and early growth response 2 (EGR2).

Based on our predictions, the TFs showing the highest contributions to gene expression throughout the brain, e.g., MAX and YY1, are involved in regulation of processes common to several cell types including metabolic processes. These results agree with the wide range of functions of MAX which have been suggested in previous studies^33^. Our approach predicted MAX as the most influential regulator in the brain. Our analysis also identified TFs regulating primarily neuronal processes such as REST. REST is considered to be the master negative regulator of neurogenesis^34, 35^.

#### Localized transcription factor contributions

Since different brain regions have different functions, an interesting aspect related to these functions is whether some TFs show brain region specific contributions, i.e., pronounced localized contributions. Similarly to region specific gene expression^20^, this tendency can be assessed by Shannon entropy for the contribution of a specific TF over the regions of the same depth (see Methods: Regression analysis of transcription factor interactions). Here, a highly region specific contribution is indicated by a low entropy whereas a high entropy indicates more evenly distributed contributions over all regions in a given brain hierarchy depth. TFs with a strong localized contribution should be characterized by a high contribution to gene expression in a region and a low entropy. We identified three TFs with strong and robust local regulatory signatures characterized by a high contribution to gene expression in one brain region and a low entropy. These TFs were in the top 1/3 quantile for the contribution in a brain region and the top 1/3 quantile for the lowest entropy over brain regions of the same depth.

Based on our results, the Enhancer of zeste 2 polycomb repressive complex 2 subunit (EZH2) showed the most localized contributions to the metencephalon and specifically to the cerebellum (Fig. 1). Our analysis suggests that EZH2 is involved in the regulation of 39 and 30 genes inside the metencephalon and its anatomical sub-region the cerebellum, respectively (Supplementary file S4). Eleven of these genes are predicted to be regulated by EZH2 in both the metencephalon and cerebellum. These eleven genes include Zic family member 2 (ZIC2) and SH3 and multiple ankyrin repeat domains (SHANK1). ZIC2 and SHANK1 are associated with nervous system development (GO-term GO:0007399). In addition, inside the cerebellum, six EZH2 regulated genes are related to neuronal processes (Supplementary file S5) including neuronal differentiation 2 (NEUROD2) and somatostatin receptor 2 (SSTR2) which are both part of the GO-terms cerebellum development (GO:0021549) and metencephalon development (GO:0022037) (Supplementary file S5). Similarly, seven additional EZH2 regulated genes inside the metencephalon are related to neuronal processes. For instance, our model predicted that EZH2 regulates neuronal PAS domain protein 3 (NPAS3).

The Nuclear receptor subfamily 3 group C member 1 (NR3C1) exhibits its most localized contributions to gene expression inside cerebral cortex (Fig. 1). In the cerebral cortex, NR3C1 is involved in the regulation of 20 genes including the ephrin receptor binding associated genes (GO:0046875) ephrin A5 (EFNA5) and cyclin dependent kinase 5 regulatory subunit 1 (CDK5R1). Both, CDK5R1 and EFNA5 are also associated with the regulation of microtubule cytoskeleton organization (GO:0070507) (Supplementary file S5). The majority of NR3C1 regulated genes (14 of the 20) are nucleus-specific (GO:0005634). The second largest contribution of NR3C1 to gene expression is inside the cerebellum (Fig. 1) where NR3C1 is involved regulating EFNA5 and CDK5R1, too.

For the serum response factor (SRF), we predicted a localized contribution to gene expression in the Basal forebrain (BF) (Supplementary file S2 and 3, Supplementary file S1, Figs S14–S18). Here, SRF contributes to the regulation of two F-box genes, F-box and leucine rich repeat protein 16 (FBXL16), and F-box protein 46 (FBXO46), SERTA domain containing 1 (SERTAD1), DEAD-box helicase 51 (DDX51) and RAB40B, member RAS oncogene family (RAB40B).

The three TFs with the most localized contributions, based on our analysis, EZH2, SRF and NR3C1 are known regulators in different contexts: EZH2 in epigenetic regulation^36^, SRF as regulator of the cytoskeleton^37^ and NR3C1 in relation to prenatal stress exposure^38^. Our results suggest additional functions of EZH2, SRF and NR3C1 in specific neuroanatomical regions.

### Regulatory elements of differentially expressed mRNAs

In addition to the analysis of TFs’ contribution to gene expression, we aimed to identify elements of mRNAs that are potentially important for post-transcriptional regulation. For this purpose, we analyzed differential gene expression between all pairs of sub-regions within nine different brain regions. Our assumption is that regulatory features in mRNAs which influence gene expression should contribute to a differential expression of the mRNAs that they are part of. In other words, for mRNAs that are not differentially expressed, there is no indication that they contain regulatory elements that influence their gene expression.

**Figure 2:**
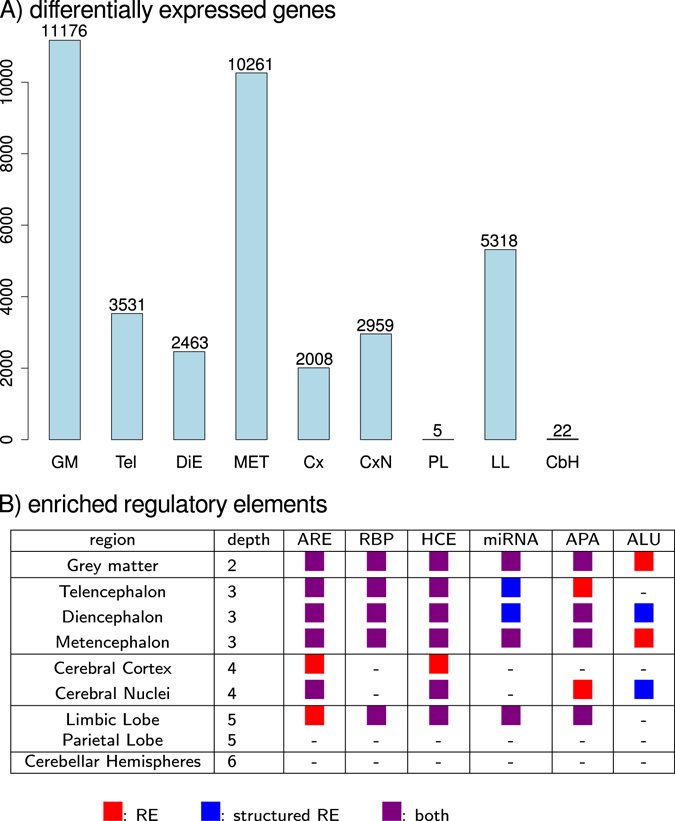
Enriched structured regulatory elements in differentially expressed genes. (**A**) The number of differentially expressed genes between any two sub-regions of the indicated region. (**B**) Enriched regulatory elements for the set of differentially expressed genes. RE refers to regulatory elements. If a RE overlaps with a RNA secondary structure predictions it is referred to as structured RE.

To address this, we analyzed regions that consist of two to five sub-regions (Supplementary file S1, Fig. S1). For regions that contained more than two sub-regions, we performed all pair-wise comparisons between sub-regions and combined all genes that were significantly differentially expressed. Differential expression analysis was performed using the limma package and a log-fold change of log_2_ 1.5 (see Methods)^39^. The number of genes that were expressed above background in each region was between 16,308 and 16,959 genes (see Methods). For the most coarse-grained level of analyzed brain regions, i.e., grey matter, we found 11,176 significantly differentially expressed genes (Fig. 2A). Similarly, there was a high number of differentially expressed genes between the cerebellum and pons inside the metencephalon, i.e., 10,261 most-likely due to a different cyto-architecture. Notably, only 5 and 22 genes were found to be differentially expressed inside the cerebellar hemispheres and parietal lobe of the cerebral cortex, respectively.

#### Enrichment of regulatory elements

For identifying Regulatory Elements (RE) of mRNAs that potentially contribute to the differential gene expression in the human brain, we computed enrichment of annotated REs located in differentially expressed genes (Supplementary file S1, Fig. S2). We considered annotated REs from the AURA database which groups REs into distinct types such as AU-rich elements (ARE), hyper-conserved elements (HCE), miRNA target sites (miRNA), RNA binding proteins binding sites (RBP) and alternative poly-adenylation sites (APA)^30^. As a prerequisite for identifying potentially regulatory RNA secondary structure motifs, we identified enriched REs that overlap with predictions for conserved RNA secondary structures. We inferred the predictions from a genome-wide screen for vertebrate-conserved RNA secondary structures (CRS) using CMfinder^31^ and computed the overlap with annotated REs from the AURA database^30^. We considered REs and structure predictions as overlapping if at least one nucleotide of an annotated RE overlaps with the genomic coordinates of the structure prediction. For simplicity, we refer to annotated REs that overlap RNA secondary structure predictions as structured REs. Differentially expressed mRNAs were considered to be enriched for types of REs or specific REs such as RBM47 binding sites if a *χ* ^2^-test resulted in a Benjamini-Hochberg adjusted p-value < 0.05 and at least ten different differentially expressed genes contained the RE.

In total, the binding sites of 55 RBPs and of 31 miRNAs were enriched in differentially expressed genes in at least one of the analyzed brain regions. In particular, structured binding sites of 32 distinct RBPs were enriched in differentially expressed genes (Supplementary file S6). However, for most cases, the binding sites of specific RBPs were also enriched when ignoring the REs overlap with RNA secondary structure predictions. We found an enrichment for structured AREs for all regions except the cerebral cortex, parietal lobe, limbic lobe and the cerebellar hemispheres (Fig. 2B). As expected by the difference in UTR lengths, fewer types of structured REs are enriched in 5′-UTRs compared with REs in 3′-UTRs (Supplementary file S1, Fig. S19). There is an enrichment for structured RBP binding sites inside 5′-UTRs of differentially expressed genes inside grey matter in general, the telencephalon, metencephalon and the limbic lobe.

#### Re-occurrence of structured regulatory elements

Our main aim in this part of the analysis is to categorize common RNA secondary structure motifs for enriched structured REs. The differentially expressed genes within any sub-region of the human brain comprise 11,363 conserved RNA secondary structures (CRS) corresponding to enriched structured REs. We searched for additional instances of the CRSs in the human genome (see Methods: Structure motif re-occurrence) by similarity of CRSs using Infernal CMsearch^40^. We identified 65 CRSs with reoccurring instances within REs of the same category in at least three different genes (see Methods: Structure motif re-occurrence). The same category refers to a specific entity of structured REs that is enriched for differentially expressed genes inside a specific region, e.g., ARE or RBP, or more specific RBP binding sites like RBM47 binding sites of differentially expressed genes inside grey matter. We required that at least 90% of the base pairs of the consensus RNA structure of a CRS were predicted for the target sequence and that the sequence did not overlap repeats except simple repeats based on RepeatMasker annotations from the UCSC Genome Browser^17^.

We further selected 15 high quality CRSs (Supplementary file S1, Figs S21–S24, Table S5,^41^) by filtering for evolutionary conservation of base pairs (normalized parsimony scores higher than median), neither extreme GC content (between 0.2–0.8) nor pair-wise sequence identity (<0.95). The evolutionary conservation of base pairs was assessed by computing parsimony scores that describe how consistently base pairs were inherited or lost inside a phylogenetic subtree (see Methods: Analysis of RNA secondary structure motifs).

**Figure 3:**
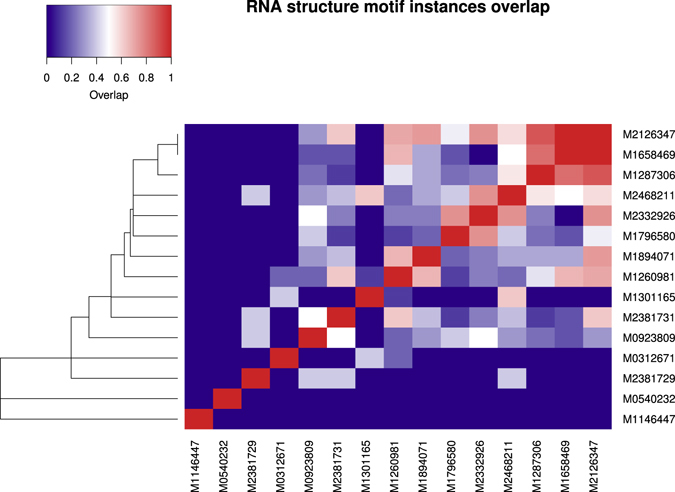
Overlap between predicted instances of reoccurring RNA structure motifs. The overlap between the predicted instances is depicted for 15 CRS motifs. The 15 CRSs are well conserved and predicted inside enriched structured regulatory elements for at least three different differentially expressed genes. The overlap between instances a CRS motif *A* and instances of a CRS motif *B* is computed by ${\rm{overlap}}=\tfrac{|A\cap B|}{{\rm{\max }}\{|A|,|B|\}}$. The heatmap shows a single-linkage hierarchical clustering between the CRS motifs.

**Figure 4:**
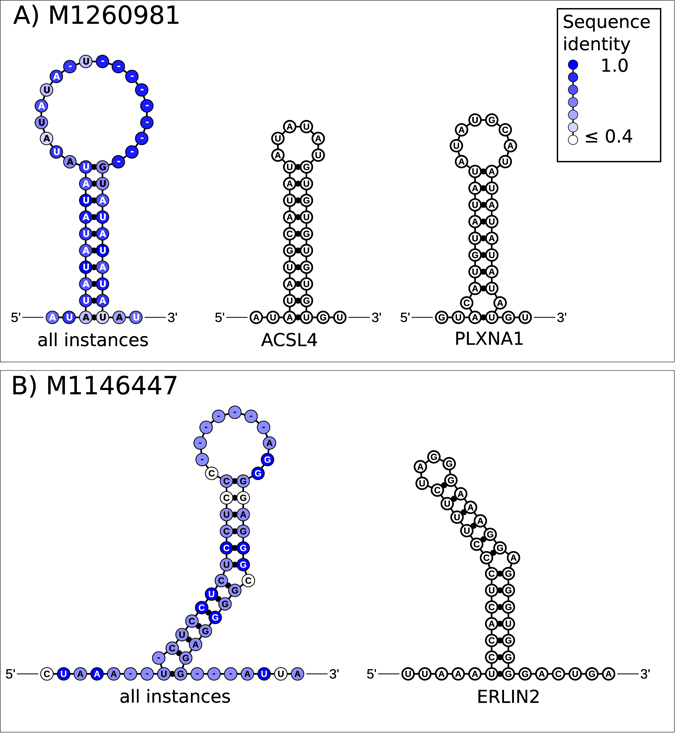
Examples of reoccurring RNA structure motifs. Each panel shows the consensus structure based on the predicted instances of the corresponding conserved RNA structure (CRS) motif and instances of the CRS in brain related genes. The sequence identity is computed over the predicted instances of the motif and given by a white to blue color gradient. RNA secondary structure diagrams were drawn with RNAfdl^104^.

We assessed the similarity of motifs corresponding to two CRS by evaluating for which sequences of structured REs they were predicted (Fig. 3). The overlap between the sequences of predicted instances was then used to compute a similarity score between 0 and 1. Sequences with predicted instances of motifs corresponding to 13 out of the 15 CRS overlap with at least one other of the 15 CRS. All 13 of these motifs contain purine-uracil tandem repeats ((RU)_*n*_ repeats). Eight of these 13 motifs form a cluster of relatively AU-rich sequences (GC content <0.3). Instances of these (RU)_*n*_ repeat motifs were predicted for several REs of neuron related genes including neuron projection associated genes (Supplementary file S7): acyl-CoA synthetase long-chain family member 4 (ACSL4), plexin A1 (PLXNA1), tetraspanin 2 (TSPAN2), Ras associated (RalGDS/AF-6) and pleckstrin homology domains 1 (RAPH1) (Fig. 4A), and potassium voltage-gated channel subfamily H member 5 (KCNCH5). See ‘Supplementary text S1 - Instances of RNA secondary structure motifs’ for additional information about CRS instances in specific genes.

The two remaining CRS motifs, which do not overlap with the other thirteen motifs, were each found in RBP binding sites of three different genes that were differentially expressed in gray matter and in the case of CRS motif M1146447 also differentially expressed inside the metencephalon ((Fig. 4B, Supplementary text S1, Figs S2 and 3C). Instances of CRS motif M1146447 were predicted in the 3′-UTRs of ER lipid raft associated 2 (ERLIN2), FK506 binding protein 5 (FKBP5) and sphingosine kinase 2 (SPHK2).

In summary, we identified a family of (RU)_*n*_-repeat RNA structure motifs and two additional structure motifs that occurred in several structured REs inside UTRs of differentially expressed genes in the brain including genes associated with neurons such as ACSL4, PLXNA1 and KCNCH5.

## Discussion

Using post-mortem gene expression data of human brains and non-brain specific ChIP-seq data, we identified the most active transcription factors (TF) in the brain and transcription factor contributions to gene expression that are localized to the metencephalon, cerebellum, cerebral cortex and Basal forebrain. In addition, we used known regulatory elements (RE) in the UTRs of mRNAs that were enriched for differentially expressed mRNAs in the brain as a starting point for finding novel RNA secondary structure motifs with potential relevance for post-transcriptional regulation.

Our results suggest that the transcription factors with the highest contributions to gene expression in all brain regions, i.e., MAX, TAF1, POLR2A, GABPA and YY1, contribute to regulating genes that are involved in more general cellular processes such as metabolic processes and secondary transcription factor activity given an enrichment for GO-terms such as nucleus and nucleic acid and chromatin binding. However, our analysis also suggests CNS and neuron related TF-gene interactions. MAX is known to interact with v-myc avian myelocytosis viral oncogene homolog (MYC), but MAX also dimerizes with several other proteins such as MAD^33^. Supporting our predictions, MAX is known to regulate genes related to cellular growth, proliferation, differentiation and metabolism. MAX is mainly active in the brain, heart and lungs^42^. For REST, we predicted the most obvious involvement in the regulation of neuron related genes including down-regulation of voltage-gated potassium channel KCNC1 and NMDA type receptor GRIN1. These results agree with observations from previous studies^34, 35^. REST is a strong repressor of neuronal gene transcription in non-neuronal cells. REST is considered to be the master negative regulator of neurogenesis. REST functions by binding to Neuron-restrictive silencer elements (NRSE) in the DNA which was identified in stem cells and different types of mature neurons. REST has been suggested to play a role in the maintenance of neuronal phenotype. REST represses genes that promote cell death in the brain and is shown to be lost in the brain in Alzheimer’s and dementia patients^43, 44^.

Although not exclusively CNS related, genes regulated by the TFs GABPA, TAF1, YY1 and POLR2A also show an enrichment for GO-terms related to localization and transport (Supplementary file S5), which might be of special relevance for neurons. CEBPB and SP1, also among the 20 TFs with the highest contributions to gene expression in all brain regions, exhibit a less diverse contribution to gene expression. Potentially related to transport in neurons, CEBPB contributes to regulation of microtubule associated genes MARK1 and MTCL1 based on our analysis. *In vivo* knock down of Mtcl1 in the cerebellum results in loss of axonal polarity in Purkinje cells. Dis-function of the gene leads to spinocerebellar ataxia. MARK1 is suggested as an autism susceptibility gene after a SNP analysis in 276 families^45, 46^. Our results indicate an involvement of SP1 in the regulation of intraciliary transport particle related genes ITF20, ITF27 and TULP3. The importance of the primary cilia in the human brain and its relation to neurological disorders has been discussed more recently^47^. Germline homozygote Tulp3 mutation resulted in death of the E14.5 day fetal mouse with defects in the hindbrain and neuronal tube^48^. Hence, the regulation of ITF20, ITF27 and TULP3 by SP1 are interesting candidate interactions for further investigation in the brain.

EZH2 showed the most localized contributions to gene expression to specific brain regions of the top three TFs, i.e., EZH2, NR3C1 and SRF. EZH2 is a member of polycomb group of genes that functions in the maintenance of the repressive state of transcription. EZH2 is known to be important for neurogenesis, controlling the renewal of progenitor cells in general and cerebellum development. Ezh2 conditional knock-out mice showed hypoplasia of the cerebellum and decreased numbers of Purkinje cells^49, 50^. Based on our analysis, EZH2 is involved in regulating NEUROD2 in the cerebellum and NPAS3 in the metencephalon, as well as SHANK1 and ZIC2 inside both the metencephalon and cerebellum among other neuron related genes. EZH2 is potentially involved in the development of ataxia-telangiectasia^51^. Mouse studies suggest that Zic2 is involved in cerebellar development, and mutations in Zic2 have been associated with sensorimotor gating abnormalities in mice^52, 53^. Related to sensorimotor gating abnormalities, SHANK1 deletions in humans have been proposed as a factor for autism spectrum disorder and in addition resulted in reduced motor functions in mice^54, 55^. A mouse study provides evidence for a role of NEUROD2 for cerebellar granule cell survival and development of ataxia^56^. NPAS3 has been identified as a risk factor for schizophrenia and bipolar disorder and inactivating mutations in NPAS1 and NPAS3 cause behavioral abnormalities in mice^57, 58^. Considering these studies, our results support the idea that abnormalities in EZH2 expression inside the cerebellum or metencephalon could be a contributing factor for the development of neurological disorders such as ataxia and autism spectrum disorder.

Based on our analysis, NR3C1 exhibits a localized contribution to gene expression in the cerebral cortex and is involved in the regulation of Ephrin A5 and CDK5R1. Supporting the role of NR3C1 in the cerebral cortex, NRC3 is a known hypothalamic-pituitary-adrenal (HPA) axis gene and has a function in controlling brain response to stress. Epigenetic regulation of NR3C1 and decreased expression in the hippocampus, a sub-region of the limbic lobe inside the cerebral cortex, has been associated with victims of child abuse^59, 60^. The gradients of Ephrin-As expression including Ephrin A5 expression were shown to effect formation of the visual map inside the cortex^61^. Abnormal expression of NR3C1 in the cerebral cortex may have a relevance for neurological disorders given the predicted contribution to regulation of CDK5R1. The accumulation p25, a cleavage product of CDK5R1, has been found to be increased in the frontal cortex of Alzheimer’s patients^62^.

For SRF, we predicted localized contribution to gene expression in the basal forebrain where SRF is predicted to regulate F-box proteins among other genes. SRF is an important TF for angiogenesis and neurodevelopmental processes. It promotes filopodia formation and contributes to normal asymmetric growth of neuritis. Srf knockout mice die early in gastrulation due to the lack of mesenchymal tissue formation including heart. Srf activation is important for regulation of neuronal migration and axon and dendrite arboration in developing cortex and retina^63^. For a discussion of SRF regulated F-box proteins refer to supplementary text S1 - Extended discussion of TF regulated genes^64–69^. Although the exact function of SRF in the brain is not obvious from our analysis, our results may suggest that SRF also provides an important contribution to gene expression in adult brains.

In addition to analyzing TF-gene interactions, we derived RNA secondary structure motifs from enriched regulatory elements located in mRNAs that are differentially expressed between different neuroanatomical regions. Here, we identified a group of hairpin motifs that have purine-uracil-repeats ((RU)_*n*_-repeats) in common. RNA secondary structures comprising trinucleotide repeats are known to be relevant to certain neurological disorders^70^. In our analysis, CRS motif M1260981 was predicted to overlap REs in five genes with neuron related functions, i.e., ACSL4, NDST1, PLXNA1, TSPAN2 and RAPH1. Mutations or deletions in ACSL4, also known as FACL4, have been previously linked to non-specific mental retardation^71^. Another motif from this group, CRS motif M2126347, was predicted in TET2 and TET3, among others genes. Tet2 activity is an important factor for epigenetic regulation during mouse brain development^72^. CRS motif M1146447, a motif lacking (RU)_*n*_-repeats, was predicted to overlap REs of ERLIN2, FKBP5 and SPHK2. ERLIN2 deletions or frame shift mutations have been implicated in spastic paraplegia and mental retardation, respectively^73, 74^. Predicted instances of M1260981 in ACSL4 and RAPH1 overlap binding sites for RBM47 (Supplemenatry text S1 - Table S5). Note that we computed an enrichment for structured binding sites for RBM47 in differentially expressed genes including these two transcripts. In contrast, NDST1, PLXNA1 and TSPAN2 are part of sets of differentially expressed transcripts that were enriched for RBP binding sites in general, but not for a specific protein. However, instances of M1260981 in NDST1, PLXNA1, TSPAN2 overlap binding sites for IGF2BP3, SRRM4 and MSI1, respectively. Instances of CRS motif M1146447 in FKBP5 overlap binding sites for RBM47 and in ERLIN2 and SPHK2 overlap binding sites for IGF2BP3. RBM47 as well as IGF2BP3, SRRM4 and MSI1 contain RNA recognition motifs (RRM). Specifically in the context of RRMs, RNA secondary structures have been suggested to enhance the affinity for RBP binding, for instance, by restricting the conformational flexibility of loop regions that contain binding motifs^75^. The double stranded-regions of the identified stem loops could also serve directly as binding motifs for RBPs. Another possibility is that the transient formation of the predicted RNA structures decreases the accessibility of single-stranded motifs. For more elaborate information on the identified CRS motifs refer to ‘Supplementary text S1 - Instances of RNA secondary structure motifs’ and ‘Supplementary text S1 - Extended discussion of RNA secondary structure motifs’. Recruited RBPs can alter the expression of a transcript in several ways. RBM47 has been suggested to be a mediator for RNA editing^76^ and to be involved in mRNA stability and splicing^77^. Differential splicing of 3′ UTRs may shorten the UTR length, thus increase the stability of a transcript, e.g. through removal of miRNA binding sites^78^. While we can only speculate about exact mechanisms, the predicted structures pose interesting candidates for potential regulatory motifs in mRNAs with relevance for CNS related processes. Further investigation of the predicted RNA secondary structure motifs is necessary to elucidate their function.

In closing, we discuss some technical aspects regarding our analysis. We designed a regression approach that focused on the most robust transcription factor interactions. As discussed in the introduction, methods that directly infer read counts or are based on TF perturbation experiments were not applicable to our study^12, 13, 18, 79^. Instead, we identified TF-gene interactions by a four step filtering process. First, we limited potential TF-gene interactions to top scoring ChIP-seq binding sites^14–17^. Second, we applied LASSO regression to model the expression of a gene as sparse linear combination of the expression of potentially interacting TFs. The LASSO penalty induces sparsity^19^, i.e., LASSO removes TFs that do not add strongly relevant information to the models. Third, we compared regression models to random models, discarding ones that did not significantly outperform random ones. A disadvantage of this third step is the randomization procedure. Random selection of training/test sets and also random gene-gene interactions both limit the reproducibility of the analysis. Therefore, in a fourth step, we only considered TF-gene interactions that were part of the linear models in five independent instances of the analysis. We initialized each instance of the analysis with different unique random seeds. Experimental factors also limit our analysis, particularly the availability of suitable ChIP-seq data from which to infer potential TF-gene interactions, as well as the post-mortem nature of the six donated brain samples^10^. We modeled gene expression based on 141 TFs that were present in the analyzed micro arrays and available from the UCSC genome browser’s ENCODE ChIP-seq tracks^17^. Consequently, this analysis may underestimate regulation by TFs, given the current estimate of ≈1500 of human TF genes^80^. Sunkin *et al*. performed a thorough quality assessment and took proper precautions for handling post-mortem tissue and data normalization when generating the data for the Allen Brain Atlas^10^. However, post-mortem RNA expression likely exhibits deviations from gene expression of functional adult brains, e.g., reduced expression of some transcripts^81^. Although we focused on robust findings in our analysis and found causal associations with brain related functions, we cannot rule out the influence of post-mortem processes on gene expression. We also would like to point out that we analyzed brain tissues of adult donors with the aim to study differences between anatomical brain regions. Consequently, insights into gene regulatory processes that drive brain tissue development are out of scope for this analysis. Similarly, the analyzed microarray data provides a snapshot of the gene expression levels. Hence, the oscillatory nature of transcriptional processes or transcriptional delay in general cannot be considered in this study^82–84^. Different studies argue in favor and against a high correlation between mRNA expression and protein expression levels^85–87^. Given a less comprehensive data set, it would be interesting to compare the results of our current models with models for which we replace TF mRNA expression levels with TF protein expression levels or ChIP-seq data.

For the second part of our study, we only focused on RNA secondary structure motifs with well conserved base pairs. We inferred conserved secondary structure predictions from a genome-wide screen using CMfinder filtered by pscore which considers phylogenetic conservation^31, 88, 89^. Subsequently, we compared the parsimony of present and absent base pairs using evolutionary subtrees containing 11 and 26 species based on 100-way genome-wide alignments, i.e., closely related primates and rodents, respectively^17, 90^. CMfinder re-aligns the input sequence alignments during structure prediction^88^. This has the advantage that our predictions are less dependent on the quality of the input alignment. We used a parsimony criterion to compare a list of candidates where we only considered structures that were predicted for approximately the same set of species. Hence, this allows us to circumvent issues associated with computing a background model or estimating mutation rates for the parsimony analysis. The separate parsimony analysis also allows us to define specific evolutionary constraints. In addition, we selected only sequences for which 90% of the base pairs of the consensus structure were conserved. Similarly to the ChIP-seq data in the first part of this analysis, the set of RBP binding sites we used was derived from CLIP-seq experiments probing specific regions or cell lines, thus they indicate potential binding sites, but give no evidence for or against functional interactions in the brain. From our analysis, it is not directly possible to deduce whether there is a functional relationship between the predicted RNA secondary structures and specific types of regulatory elements. The contribution of the predicted RNA secondary structures to gene expression has to be proved experimentally, for instance by knockout experiments. Nonetheless, we identified promising candidate CRS motifs that may have functional implication for gene expression in brain regions.

## Conclusion

We identified TF-gene interactions and RNA secondary structure motifs in mRNAs that facilitate potentially important functions in the CNS and could be relevant to the etiology of neurological disorders. In particular, RNA secondary structure motifs identified in ERLIN1, ACSL4, TET2 and TET3, as well as the localized contribution of EZH2 to the regulation of ZIC2 and SHANK1 in the cerebellum are promising candidate for future investigation. Experiments such as protein-binding assays, RNA structure probing and mutation studies, immuno-precipitation approaches or knockout studies could help to elucidate the underlying processes and to verify functional implications.

## Methods

### Data sets

#### Allen Human Brain Atlas

Whole-genome micro array data of post-mortem human brain region was downloaded from the Allen Brain Atlas for six human donors^10^. See Supplementary file S1: Micro array data sets, page 3–5, for a more elaborate description of the data set. We only used micro array data instead of RNA-seq data as the whole brain micro array data is available for six donors, but the RNA-seq data only for two donors. Hence, the RNA-seq data would provide less robust statistics. The micro array data is log-normalized and contains present-call information. The data set contains between ≈300–890 samples for each of the donors covering all anatomical structures inside the brain. The data set contains samples for the left brain hemisphere for all donors and both brain hemispheres for left-handed donors. To reduce the bias of samples originating from different human donors, only samples of left brain hemispheres were selected. When analyzing specific brain regions, we combined all samples of all brain sub-regions of the corresponding region using the ontology as specified by the Allen Brain Atlas. For robustness, we limited the analysis to brain regions that contain at least 20 samples summed up over all donors after collapsing replicates separately for each donor. This yields test sets that contain two or more samples for a 10-fold cross-validation that is performed as part of the analysis of transcription factor interactions.

#### Transcription factor interactions

Transcription factor (TF) binding site predictions were obtained from the UCSC Genome Browser ENCODE ChIP-seq track^14–17^. HGNC gene symbols of transcription factors were mapped to Entrez GeneID using ENSEMBL Biomart^91^. In the following, we describe how we obtained TF-gene interaction pairs. The UCSC ENCODE ChIP-seq track contains scores for clusters of binding sites for each TF. We converted these scores into Z-scores for each TF separately and selected only TF binding site predictions that achieved a Z-score of at least 1.96. If a TF binding site was within ±10,000 nucleotides distance to the transcription start site (TSS) of a gene we assigned a TF-target gene pair. This corresponds to interactions up- and downstream of the TSS that are assumed to have a moderate effect on the target gene expression^92^. The resulting set of TF-target gene pairs contains 256,852 interactions between 141 TFs and 14,309 genes that overlap with Entrez gene IDs from the analyzed micro array data.

#### Conserved RNA secondary structure predictions

Conserved RNA secondary structures (CRSs) were inferred from a genome-wide screen for conserved RNA secondary structures^31^. To briefly summarize the methods of the screen, RNA secondary structures were predicted using CMfinder with a maximum base pair span for a single stem loop of 100 nt, and otherwise default parameters and pscore ≥50^88, 89^. CMfinder generates a covariance model (CM), a probabilistic representation of the structure and evolutionary information, for each predicted CRS. Initial CRS predictions were performed by Seemann *et al*. using sequences from the 17-way MULTIZ alignment (MA) blocks^93^. The structural alignments were then extended by searching their CMs in orthologous sequences in each of the 100 vertebrate genomes in UCSC’s 100-species alignment using the Infernal framework^17, 31, 40^.

#### AURA

Regulatory elements (REs) were obtained from the AURA database^30^. The AURA database contains a collection of RNA protein binding sites from various CLIP-seq and similar data sets, validated miRNA target sites from miRecords and mirTarbase, AU-rich elements from the AREsite database and REs from literature mining and additional sources^94–97^. Since AURA only provides UTR specific coordinates for REs, we limited the analysis to REs in UTRs that mapped directly to genomic coordinates, e.g., we excluded spliced UTRs. The UCSC IDs of corresponding genes as provided by AURA were mapped to EntrezGeneIDs using Biomart^91^. In addition, we computed the overlap of REs provided by AURA and the predicted RNA secondary structures (see above) based on their genomic coordinates.

### Regression analysis of transcription factor-gene interactions

A regression analysis was performed for evaluating TF-gene interactions inside each brain region separately (Supplementary file S1, Fig. S3). Only probes were considered for which at least 50% of the samples had a present call. We collapsed replicated samples by their median expression values and then collapsed probes that referred to the same EntrezGeneID by the median of expression value since we do not pool variances and replicated samples or probes may affect the weights of coefficients. In addition, we performed a cross-validation (CV). Replicates could compromise the validity of the CV.

We modeled the regulation by TFs of a gene *g* as the following regression problem, i.e., the expression of a gene *g* is modeled as a linear combination of the expression of transcription factors that target gene *g*:

1 $${y}_{g}={X}_{tf}\beta +\varepsilon $$

where *y*_*g*_ is a (*n*_*d*_ × 1)-vector that contains the expression of gene *g*. *n*_*d*_ is the number of samples and *X*_*tf*_ is an (*n*_*d*_ × *n*_*tf*_)-matrix that contains the expression of each TF that is predicted to interact with target gene *g* (see Data sets: Transcription factor interactions). *n*_*tf*_ is the number of TFs that are predicted to interact with gene *g*. *β* and *ε* refer to the coefficients to be determined and residuals, respectively. The sum of squared errors (SSE) of the fit, also known as residual sum of squares (RSS), is then defined as:

2 $${\rm{SSE}}={\Vert \varepsilon \Vert }_{{l}_{2}}^{2}={({y}_{g}-{X}_{tf}\beta )}^{{\rm{\top }}}({y}_{g}-{X}_{tf}\beta )$$

${\Vert \ldots \Vert }_{{l}_{2}}$ refers to the *l*_2_-norm. Regression analysis computes a fit, i.e., the coefficients *β*, to minimize the SSE. For each brain region, we first performed a least absolute shrinkage and selection operator (LASSO) regression using the glmnet R package^19, 98^. LASSO regression introduces sparsity, i.e., a subset of the coefficients *β* are set to zero, by adding a *l*_1_-norm regularization term to the minimization problem. The following minimization problem is solved for LASSO regression:

3 $$\mathop{{\rm{\min }}}\limits_{\beta }\{\frac{1}{2{n}_{d}}{\Vert {y}_{g}-{X}_{tf}\beta \Vert }_{{l}_{2}}^{2}+\lambda {\Vert \beta \Vert }_{{l}_{1}}\}$$

${\Vert \ldots \Vert }_{{l}_{1}}$ refers to the *l*_1_-norm. The regularization parameter *λ* was determined using the ‘cv.glmnet’ procedure of the glmnet R package with the one-standard-error-rule. Next, we selected only TFs with non-zero coefficients and at most *n*_*d*_ − 1 coefficients. The *X*_*tf*_ matrix was then reduced to the expression data of the selected TFs and we performed a 10-fold cross validation (CV) for fitting a linear model using the R function ‘lm’. For each fold of the CV, we computed the SSE for the fit. Since LASSO regression requires at least two parameters no LASSO regression was performed in advance if fewer than two TFs were predicted to target a gene *g*. For evaluating the regression models, we generated ten random models for each gene where we picked *n*_*tf*_ random genes instead of the TFs predicted to target the gene and performed the same procedure including LASSO regression as mentioned above. We defined that a gene was regulated by TFs if the average SSE was significantly lower than the average SSE of the random models. To evaluate whether the average SSE was significantly lower than the one of random models, we performed one-sided t-tests where we compared the 10 SSEs of the original models with the 100 SSEs of the random models. Here, the null hypothesis is that the SSEs between the TF-interaction based models and random models follows the same distribution. The alternative hypothesis is that the SSEs are smaller than the ones of random models. The resulting p-values were adjusted over all genes using the ‘p.adjust’ R function with a Benjamini-Hochberg correction^99^. We considered an adjusted p-value of 0.05 significant. In the following, only significant TF-gene interactions are used.

For evaluating the influence of each TF towards the expression of a predicted target gene, we computed the relative contribution of each TF to a target gene based on the computed LASSO coefficients. Here, we first compute the contribution of each TF for each sample:

4 $${c}_{tf,g,i}=\frac{{x}_{tf,i}\cdot {\beta }_{tf}}{{y}_{g,i}}$$

Here, *β*_*tf*_ is the LASSO coefficient of a single TF, *x*_*tf*,*i*_ and *y*_*g*,*i*_ refer to the expression of the TF and target gene of sample *i*, respectively. The TF contribution *c*_*tf*,*g*_ for a gene *g* expressed inside a region is then defined as the mean value over the set of samples *D*:

5 $${c}_{tf,g}=\frac{{\sum }_{i\in D}{c}_{tf,g,i}}{|D|}$$

The overall contribution of a TF *tf* for a region is given by the absolute sum over the contributions to all genes *g*:

6 $${s}_{tf}={\sum }_{g}|{c}_{tf,g}|$$

We ranked all TFs by their absolute sum of relative contributions *s*_*tf*_ inside each region separately. To identify TFs that exhibit high or low localized contributions to gene expression, we calculated Shannon entropy over all regions inside the same brain region ontology coarse-graining level which we refer to as depth. Our approach essentially follows the definition for region specific gene expression by Schug *et al*.^20^. We define the relative frequency *p*_*tf*,*k*_ for a specific transcription factor *tf* in region $k\in T$ by:

7 $${p}_{tf,k}=\frac{{s}_{tf,k}}{{\sum }_{j\in T}{s}_{tf,j}}$$

where *T* refers to the set of regions, *s*_*tf*,*k*_ and *s*_*tf*,*j*_ refer to the absolute sum of contributions of the TF for region *k* and region *j*, respectively. Consequently, the Shannon entropy for the contribution of a TF *tf* is given by:

8 $${e}_{tf}=-\sum _{j\in T}\,{p}_{tf,j}\,\mathrm{log}\,({p}_{tf,j})$$

A low entropy indicates a TF with stronger local contributions to one or a few regions while high entropies indicate more evenly distributed contributions over the regions.

Two steps during this procedure involve randomization, i.e., choosing the *λ* value during LASSO regression and the comparison of linear models to random models. To consider random artifacts and the reproducibility of our results, we defined a list of 500 unique random numbers and repeated the same analysis five times. Here, we used each time and for each region a different random number from the list of random numbers for initializing the random number generator.

### Differential expression analysis

We computed the pair-wise differential expression between all sub-regions of a specified brain region (Supplementary file S1, Fig. S1). This was done from a brain region ontology depth 2 down to a brain region ontology depth 6. In total, this includes nine different brain regions and pair-wise comparisons between two to five corresponding sub-regions (Supplementary file S1, Table S4). For the differential expression analysis, only probes were considered for which at least 50% of the samples of at least one of the sub-region had a present call. Considering the post-mortem nature of the heterogeneous regions and expected noise, pair-wise differential expression analysis was performed using limma with a fold-change threshold of log_2_ 1.5^39^. As recommended by the limma user-guide, the TREAT procedure with fold-change threshold was used^100^. Afterwards, ‘decideTests’ was used for the selection of significantly differentially expressed genes. The null hypothesis in this case is that the difference in mean expression between two regions is within a fold-change threshold of log_2_ 1.5. Benjamini-Hochberg corrections for multiple testing was applied to each contrast separately^99^, but samples of all sub-regions of an analyzed brain region were used as input at once. A design matrix was defined in such a way that all samples of the same sub-region were assigned to the same source. In contrast to the regression analysis, we did not collapse replicates since procedures such as limma fit a linear model for pooling variances, thus replicates are already considered and provide additional information. All genes that were differentially expressed between any two sub-regions were collected as the set of differentially expressed genes for a specific brain region. In addition, based on significant up- and down-regulation over all contrasts, we identified probes that had a significantly higher or lower expression inside one sub-region compared to all sub-regions. We refer to such genes as genes with localized expression in a specific brain region.

### Enrichment analysis of regulatory elements

For analyzing the enrichment between REs and differentially expressed genes, we defined the set of all protein-coding genes by ENSEMBL transcripts that could be mapped to EntrezGeneIDs^91, 101^. Chi-square test were then used to evaluate the association between differentially expressed genes and genes that contain REs inside their UTRs (Supplementary file S1, Fig. S2). For this purpose, the R function ‘chisq.test’ was used. The R function ‘chisq.test’ estimates p-values through a Monte Carlo approximation. We used 10^8^ replicates for the Monte Carlo approximation as this number of replicates provided stable p-values up to the third decimal place. Here, we only considered REs that were present in the UTRs of at least ten differentially expressed genes and ten genes that were not differentially expressed to ensure a relevant approximation of p-values. We distinguished between RE types such as AU-rich elements (ARE) or RNA binding proteins (RBP) and specific RE elements such as ELAVL1 bindings sites or miR-96 target sites. For each region separately, we performed Chi-square tests for REs and structured REs (see above). We then performed Benjamini-Hochberg corrections for multiple-testing separately for RE types and RE elements for each region using the R function ‘p.adjust’^99^. An adjusted p-value of 0.05 was considered significant.

### Analysis of RNA secondary structure motifs

#### Conserved phylogenetic subtrees and filtering

For assessing the quality of a predicted RNA secondary structure, we inspected evolutionary conservation of the predicted structure. We developed an approach to identify the largest phylogenetic subtree with respect to the number of species inside a phylogenetic tree of 100 vertebrate species for which the structure motif was predicted for at least 80% of the species. For the structure predictions^31^ (see Data sets: Conserved RNA secondary structure predictions), we computed fingerprints (FP) that indicate presence and absence of base pairs of the consensus structure by projecting the consensus structure to the individual sequences. A base pair is absent if the projection resulted in non-canonical base pairs or included gaps. Leafs in a phylogenetic tree are annotated with the corresponding fingerprints for each species. Next, we identified the largest phylogenetic subtree where the FP of each leaf had a dissimilarity to the human FP smaller than a defined threshold ${\theta }_{{d}_{h}}=0.2$. Therefore, we computed Hamming distances *h* between FPs and calculated a ratio ${d}_{h}=\tfrac{h}{{n}_{bp}}$ where *n*_*bp*_ is the number of base pairs of the consensus structure. The largest phylogenetic sub-tree is identified by iteratively processing the subtree of the next common ancestor of human. Note that we used the 100 vertebrate phylogenetic tree given as New Hampshire (NH) standard, i.e., in Newick tree format, from the UCSC genome browser where all ancestor nodes are internal nodes and not assigned to a specific species^17^. For each iteration, the fraction of species that contains a FP with a sufficient similarity to the human FP is evaluated. In this way, we identified the largest phylogenetic subtree in which the predicted structure motif is conserved for at least 80% of the species. We computed the average pair-wise sequence identity and the GC content over the sequences of the resulting phylogenetic subtree. To compute the average pair-wise sequence identity, we counted identities between non-gap characters of each two sequences normalized by the gap-free length of the shorter sequence and computed the average of all pair-wise comparisons. For the selection of reoccurring motifs, only motifs with a sequence identity lower than 95% and a GC content, *c*_*gc*_, in a range 0.2 < *c*_*gc*_ < 0.8 were considered. Sequences that overlapped non-simple repeats annotated in the UCSC GenomeBrowser *rmask* track (hg38) by RepeatMasker were removed from this part of the analysis (Smit, AFA, Hubley, R and Green, P. RepeatMasker Open-4.0. 2013–2015, http://www.repeatmasker.org)^17, 102, 103^.

#### Parsimony computation

To select evolutionarily well conserved candidate structures, we calculated parsimony scores inside different subtrees for the base pair fingerprints (FP). Parsimony scores indicate the minimum number of necessary relabeling operations for each base pair given the phylogenetic tree. Different subtrees were defined based on branch lengths in the phylogenetic tree that we obtain from the UCSC genome browser^17^. We defined five subtrees that comprise i) the 11 most closely related primates *T*_11_, ii) 26 primates and rodents *T*_26_, iii) the 51 most closely related mammals *T*_51_, iv) 62 mammals *T*_62_ and v) 82 mammals, lizards and birds *T*_82_ (Supplementary file S1, Fig. S1). Parsimony scores were calculated in a bottom-up manner as specified by the Fitch algorithm^90^. In case of a missing sequence for a species, i.e., when CMsearch did not return a hit for the corresponding sequence of the species, no penalty was added, but we limited the parsimony computation to RNA secondary structures motifs that were predicted for 80% of the species inside the analyzed subtree (see last section). The parsimony score was then weighted by the sequence length and the number of species. For FPs the alphabet is ${\mathscr{A}}=\{0,1\}$ where 0 indicates the absence and 1 the presence of the base pair. Motifs were selected that exhibited a FP based parsimony score lower than the median parsimony score of all RNA secondary structure motifs for both subtree *T*_11_ and subtree *T*_26_.

#### Structure motifs reoccurrence

From the structural alignment of each CRS that overlapped annotated enriched regulatory elements (structured REs) in differentially expressed genes, we extracted the human sequence and extended it 25 nt upstream and 25 nt downstream of the predicted structure. CMsearch was then used to identify significantly similar motifs in all human sequences containing enriched structured REs, i.e., CMsearch hits with a significant E-value indicated by a exclamation mark in the output^40^. The covariance models (CM) of the RNA secondary structure motifs were calibrated on the 17-way structural alignments that were used for the initial predictions. For predicted instances of a motif, we verified that 80% of the base pairs of the consensus structure could be successfully projected to the matched sequence, as mentioned above. From this set, we selected instances of the motifs that were found in enriched structured REs of genes which were differentially expressed in a given brain region, for each region separately.

### GO-term enrichment analysis

GO-term enrichment for genes was computed using the *over*-*representation analysis* feature of the ConsensusPathDB web server (release 31) with default parameters^32^. For differentially expressed genes, we defined the background as all genes that were part of the analyzed micro arrays and annotated with EntrezGeneIDs. The ConsensusPathDB computes enrichment through a hyper-geometric test and applies a false discovery rate (FDR) correction. GO terms with a q-value < 0.05 were considered significant for this study.

## Electronic supplementary material

S1 Supplementary text

Supplementary Dataset Number: S2

Supplementary Dataset Number: S3

Supplementary Dataset Number: S4

Supplementary Dataset Number: S5

Supplementary Dataset Number: S6

Supplementary Dataset Number: S7

## References

[1] Maston GA, Evans SK, Green MR (2006). Transcriptional regulatory elements in the human genome. Annu Rev Genomics Hum Genet.

[2] Wu X, Brewer G (2012). The regulation of mRNA stability in mammalian cells: 2.0. Gene.

[3] Black DL (2003). Mechanisms of alternative pre-messenger RNA splicing. Annu Rev Biochem.

[4] Gerstberger S, Hafner M, Tuschl T (2014). A census of human RNA-binding proteins. Nat Rev Genet.

[5] Meunier, D., Lambiotte, R. & Bullmore, E. T. Modular and hierarchically modular organization of brain networks. *Front Neurosci* **4**, doi:10.3389/fnins.2010.00200 (2010). 10.3389/fnins.2010.00200 PMC3000003 21151783

[6] Brooks, V. B. & Thach, W. T. *Handbook of physiology*, *The Nervous System*, *Motor Control*, chap. Cerebellar control of posture and movement, 877–946 (American Physiological Society, Bethesda, 1981).

[7] Strick PL, Dum RP, Fiez JA (2009). Cerebellum and nonmotor function. Annu Rev Neurosci.

[8] Jonides J (2008). The mind and brain of short-term memory. Annu Rev Psychol.

[9] Azevedo FAC (2009). Equal numbers of neuronal and nonneuronal cells make the human brain an isometrically scaled-up primate brain. J Comp Neurol.

[10] Sunkin SM (2013). Allen Brain Atlas: an integrated spatio-temporal portal for exploring the central nervous system. Nucleic Acids Res.

[11] Hawrylycz MJ (2012). An anatomically comprehensive atlas of the adult human brain transcriptome. Nature.

[12] Gao, F., Foat, B. C. & Bussemaker, H. J. Defining transcriptional networks through integrative modeling of mRNA expression and transcription factor binding data. *BMC Bioinformatics* **5**, doi:10.1186/1471-2105-5-31 (2004). 10.1186/1471-2105-5-31 PMC407845 15113405

[13] Ouyang Z, Zhou Q, Wong WH (2009). ChIP-Seq of transcription factors predicts absolute and differential gene expression in embryonic stem cells. Proc Natl Acad Sci USA.

[14] Wang J (2012). Sequence features and chromatin structure around the genomic regions bound by 119 human transcription factors. Genome Res.

[15] Wang J (2013). Factorbook.org: a Wiki-based database for transcription factor-binding data generated by the ENCODE consortium. Nucleic Acids Res.

[16] Gerstein MB (2012). Architecture of the human regulatory network derived from ENCODE data. Nature.

[17] Speir ML (2016). The UCSC Genome Browser database: 2016 update. Nucleic Acids Res.

[18] Haynes BC (2013). Mapping functional transcription factor networks from gene expression data. Genome Res.

[19] Tibshirani R (1994). Regression Shrinkage and Selection Via the Lasso. Journal of the Royal Statistical Society, Series B.

[20] Schug, J. *et al*. Promoter features related to tissue specificity as measured by Shannon entropy. *Genome Biol* **6**, doi:10.1186/gb-2005-6-4-r33 (2005). 10.1186/gb-2005-6-4-r33 PMC1088961 15833120

[21] Leppek K (2013). Roquin promotes constitutive mRNA decay via a conserved class of stem-loop recognition motifs. Cell.

[22] Seemann, S. E., Sunkin, S. M., Hawrylycz, M. J., Ruzzo, W. L. & Gorodkin, J. Transcripts with in silico predicted RNA structure are enriched everywhere in the mouse brain. *BMC Genomics* **13**, doi:10.1186/1471-2164-13-214 (2012). 10.1186/1471-2164-13-214 PMC3464589 22651826

[23] Rabani M, Kertesz M, Segal E (2008). Computational prediction of RNA structural motifs involved in posttranscriptional regulatory processes. Proc Natl Acad Sci USA.

[24] Ray D (2009). Rapid and systematic analysis of the RNA recognition specificities of RNA-binding proteins. Nat Biotechnol.

[25] Kazan, H., Ray, D., Chan, E. T., Hughes, T. R. & Morris, Q. RNAcontext: a new method for learning the sequence and structure binding preferences of RNA-binding proteins. *PLoS Comput Biol* **6**, doi:10.1371/journal.pcbi.1000832 (2010). 10.1371/journal.pcbi.1000832 PMC2895634 20617199

[26] Maticzka, D., Lange, S. J., Costa, F. & Backofen, R. GraphProt: modeling binding preferences of RNA-binding proteins. *Genome Biol* **15**, doi:10.1186/gb-2014-15-1-r17 (2014). 10.1186/gb-2014-15-1-r17 PMC4053806 24451197

[27] Ray D (2013). A compendium of RNA-binding motifs for decoding gene regulation. Nature.

[28] Gardner, P. P. & Giegerich, R. A comprehensive comparison of comparative RNA structure prediction approaches. *BMC Bioinformatics* **5**, doi:10.1186/1471-2105-5-140 (2004). 10.1186/1471-2105-5-140 PMC526219 15458580

[29] Puton T, Kozlowski LP, Rother KM, Bujnicki JM (2013). CompaRNA: a server for continuous benchmarking of automated methods for RNA secondary structure prediction. Nucleic Acids Res.

[30] Dassi, E. *et al*. AURA 2: Empowering discovery of post-transcriptional networks. *Translation* (*Austin*) **2**, doi:10.4161/trla.27738 (2014). 10.4161/trla.27738 PMC4705823 26779400

[31] Seemann, S. E. *et al*. The identification and functional annotation of RNA structures conserved in vertebrates. *Genome Res* **Published in Advance**, doi:10.1101/gr.208652.116 (2017). 10.1101/gr.208652.116 PMC5538553 28487280

[32] Kamburov A, Stelzl U, Lehrach H, Herwig R (2013). The ConsensusPathDB interaction database: 2013 update. Nucleic Acids Res.

[33] Link JM, Hurlin PJ (2015). The activities of MYC, MNT and the MAX-interactome in lymphocyte proliferation and oncogenesis. Biochim Biophys Acta, Gene Regul Mech.

[34] Cheong A (2005). Downregulated REST transcription factor is a switch enabling critical potassium channel expression and cell proliferation. Mol Cell.

[35] Noh K-M (2012). Repressor element-1 silencing transcription factor (REST)-dependent epigenetic remodeling is critical to ischemia-induced neuronal death. Proc Natl Acad Sci USA.

[36] Viré E (2006). The Polycomb group protein EZH2 directly controls DNA methylation. Nature.

[37] Miano JM, Long X, Fujiwara K (2007). Serum response factor: master regulator of the actin cytoskeleton and contractile apparatus. Am J Physiol: Cell Physiol.

[38] Oberlander TF (2008). Prenatal exposure to maternal depression, neonatal methylation of human glucocorticoid receptor gene (NR3C1) and infant cortisol stress responses. Epigenetics.

[39] Ritchie, M. E. *et al*. limma powers differential expression analyses for RNA-sequencing and microarray studies. *Nucleic Acids Res* **43**, doi:10.1093/nar/gkv007 (2015). 10.1093/nar/gkv007 PMC4402510 25605792

[40] Nawrocki EP, Eddy SR (2013). Infernal 1.1: 100-fold faster RNA homology searches. Bioinformatics.

[41] Bernhart, S. H., Hofacker, I. L., Will, S., Gruber, A. R. & Stadler, P. F. RNAalifold: improved consensus structure prediction for RNA alignments. *BMC bioinformatics* **9**, doi:10.1186/1471-2105-9-474 (2008). 10.1186/1471-2105-9-474 PMC2621365 19014431

[42] Ecevit O, Khan MA, Goss DJ (2010). Kinetic analysis OF B/HLH/Z transcription factors MYC, MAX and MAD interaction with cognate DNA. Biochemistry.

[43] Schoenherr CJ, Anderson DJ (1995). The neuron-restrictive silencer factor (NRSF): a coordinate repressor of multiple neuron-specific genes. Science.

[44] Lu T (2014). REST and stress resistance in ageing and Alzheimer’s disease. Nature.

[45] Satake T (2017). MTCL1 plays an essential role in maintaining Purkinje neuron axon initial segment. EMBO J.

[46] Maussion G (2008). Convergent evidence identifying MAP/microtubule affinity-regulating kinase 1 (MARK1) as a susceptibility gene for autism. Human Mol Genet.

[47] Guemez-Gamboa A, Coufal NG, Gleeson JG (2014). Primary cilia in the developing and mature brain. Neuron.

[48] Ikeda A, Ikeda S, Gridley T, Nishina PM, Naggert JK (2001). Neural tube defects and neuroepithelial cell death in Tulp3 knockout mice. Human Mol Genet.

[49] Akizu, N. *et al*. EZH2 regulates neuroepithelium structure and neuroblast proliferation by repressing p21. *Open Biol* **6**, doi:10.1098/rsob.150227 (2016). 10.1098/rsob.150227 PMC4852452 27248655

[50] Feng X (2016). Polycomb Ezh2 controls the fate of GABAergic neurons in the embryonic cerebellum. Development.

[51] Li J (2013). EZH2-mediated H3K27 trimethylation mediates neurodegeneration in ataxia-telangiectasia. Nature neuroscience.

[52] Aruga J, Inoue T, Hoshino J, Mikoshiba K (2002). Zic2 controls cerebellar development in cooperation with Zic1. J Neurosci.

[53] Ogura H, Aruga J, Mikoshiba K (2001). Behavioral abnormalities of Zic1 and Zic2 mutant mice: implications as models for human neurological disorders. Behav Genet.

[54] Sato D (2012). SHANK1 Deletions in Males with Autism Spectrum Disorder. Am J Hum Genet.

[55] Silverman JL (2011). Sociability and motor functions in Shank1 mutant mice. Brain Res.

[56] Olson JM (2001). NeuroD2 is necessary for development and survival of central nervous system neurons. Dev Biol.

[57] Pickard BS (2009). Interacting haplotypes at the NPAS3 locus alter risk of schizophrenia and bipolar disorder. Mol Psychiatry.

[58] Erbel-Sieler C (2004). Behavioral and regulatory abnormalities in mice deficient in the NPAS1 and NPAS3 transcription factors. Proc Natl Acad Sci USA.

[59] McGowan PO (2009). Epigenetic regulation of the glucocorticoid receptor in human brain associates with childhood abuse. Nat Neurosci.

[60] Pagliaccio D (2015). HPA axis genetic variation, pubertal status, and sex interact to predict amygdala and hippocampus responses to negative emotional faces in school-age children. Neuroimage.

[61] Cang J (2005). Ephrin-as guide the formation of functional maps in the visual cortex. Neuron.

[62] Tseng HC, Zhou Y, Shen Y, Tsai LH (2002). A survey of Cdk5 activator p35 and p25 levels in Alzheimer’s disease brains. FEBS Lett.

[63] Scandaglia, M. *et al*. Fine-tuned SRF activity controls asymmetrical neuronal outgrowth: implications for cortical migration, neural tissue lamination and circuit assembly. *Sci Rep* **5**, doi:10.1038/srep17470 (2015). 10.1038/srep17470 PMC4671020 26638868

[64] Nelson, D. E., Randle, S. J. & Laman, H. Beyond ubiquitination: the atypical functions of Fbxo7 and other F-box proteins. *Open Biol* **3**, doi:10.1098/rsob.130131 (2013). 10.1098/rsob.130131 PMC3814724 24107298

[65] Di Fonzo A (2009). FBXO7 mutations cause autosomal recessive, early-onset parkinsonian-pyramidal syndrome. Neurology.

[66] Gong B (2010). SCFFbx2-E3-ligase-mediated degradation of BACE1 attenuates Alzheimer’s disease amyloidosis and improves synaptic function. Aging Cell.

[67] Knoell B, Nordheim A (2009). Functional versatility of transcription factors in the nervous system: the SRF paradigm. Trends Neurosci.

[68] Stritt C (2009). Paracrine control of oligodendrocyte differentiation by SRF-directed neuronal gene expression. Nat Neurosci.

[69] Gelfand, Y. & Kaplitt, M. G. Gene therapy for psychiatric disorders. *World Neurosurg* **80**, doi:10.1016/j.wneu.2012.12.028 (2013). 10.1016/j.wneu.2012.12.028 23268195

[70] Sobczak K, de Mezer M, Michlewski G, Krol J, Krzyzosiak WJ (2003). RNA structure of trinucleotide repeats associated with human neurological diseases. Nucleic Acids Res.

[71] Meloni I (2002). FACL4, encoding fatty acid-CoA ligase 4, is mutated in nonspecific X-linked mental retardation. Nature Genet.

[72] Lister, R. *et al*. Global epigenomic reconfiguration during mammalian brain development. *Science* **341**, doi:10.1126/science.1237905 (2013). 10.1126/science.1237905 PMC3785061 23828890

[73] Alazami AM, Adly N, Al Dhalaan H, Alkuraya FS (2011). A nullimorphic ERLIN2 mutation defines a complicated hereditary spastic paraplegia locus (SPG18). Neurogenetics.

[74] Yildirim Y (2011). A frameshift mutation of ERLIN2 in recessive intellectual disability, motor dysfunction and multiple joint contractures. Hum Mol Genet.

[75] Maris C, Dominguez C, Allain FH-T (2005). The RNA recognition motif, a plastic RNA-binding platform to regulate post-transcriptional gene expression. FEBS J.

[76] Fossat N (2014). C to U RNA editing mediated by APOBEC1 requires RNA-binding protein RBM47. EMBO Rep.

[77] Vanharanta, S. *et al*. Loss of the multifunctional RNA-binding protein RBM47 as a source of selectable metastatic traits in breast cancer. *Elife* **3**, doi:10.7554/eLife.02734 (2014). 10.7554/eLife.02734 PMC4073284 24898756

[78] Matoulkova E, Michalova E, Vojtesek B, Hrstka R (2012). The role of the 3’untranslated region in post-transcriptional regulation of protein expression in mammalian cells. RNA Biol.

[79] Foat BC, Morozov AV, Bussemaker HJ (2006). Statistical mechanical modeling of genome-wide transcription factor occupancy data by MatrixREDUCE. Bioinformatics.

[80] Wingender E, Schoeps T, Haubrock M, Dönitz J (2015). TFClass: a classification of human transcription factors and their rodent orthologs. Nucleic Acids Res.

[81] Ferrer, I., Martinez, A., Boluda, S., Parchi, P. & Barrachina, M. Brain banks: benefits, limitations and cautions concerning the use of post-mortem brain tissue for molecular studies. *Cell Tissue Bank* **9**, doi:10.1007/s10561-008-9077-0 (2008). 10.1007/s10561-008-9077-0 18543077

[82] Monk NA (2003). Oscillatory expression of Hes1, p53, and NF-*κ*B driven by transcriptional time delays. Curr Biol.

[83] Shimojo H (2016). Oscillatory control of Delta-like1 in cell interactions regulates dynamic gene expression and tissue morphogenesis. Genes Dev.

[84] Honkela A (2015). Genome-wide modeling of transcription kinetics reveals patterns of RNA production delays. Proc Natl Acad Sci USA.

[85] Li, J. J., Bickel, P. J. & Biggin, M. D. System wide analyses have underestimated protein abundances and the importance of transcription in mammals. *PeerJ* **2**, doi:10.7717/peerj.270 (2014). 10.7717/peerj.270 PMC3940484 24688849

[86] Liu Y, Beyer A, Aebersold R (2016). On the dependency of cellular protein levels on mRNA abundance. Cell.

[87] Jovanovic, M. *et al*. Dynamic profiling of the protein life cycle in response to pathogens. *Science* **347**, doi:10.1126/science.1259038 (2015). 10.1126/science.1259038 PMC4506746 25745177

[88] Yao Z, Weinberg Z, Ruzzo WL (2006). CMfinder–a covariance model based RNA motif finding algorithm. Bioinformatics.

[89] Yao, Z. *Genome scale search of noncoding RNAs*: *Bacteria to Vertebrates*. Ph.D. thesis, University of Washington, Seattle, WA (2008).

[90] Fitch WM (1971). Toward Defining the Course of Evolution: Minimum Change for a Specific Tree Topology. Syst Zool.

[91] Kinsella, R. J. *et al*. Ensembl BioMarts: a hub for data retrieval across taxonomic space. *Database* (*Oxford*) **2011**, doi:10.1093/database/bar030 (2011). 10.1093/database/bar030 PMC3170168 21785142

[92] MacIsaac, K. D. *et al*. A quantitative model of transcriptional regulation reveals the influence of binding location on expression. *PLoS Comput Biol* **6**, doi:10.1371/journal.pcbi.1000773 (2010). 10.1371/journal.pcbi.1000773 PMC2861697 20442865

[93] Blanchette M (2004). Aligning multiple genomic sequences with the threaded blockset aligner. Genome Res.

[94] Dassi E (2012). AURA: Atlas of UTR Regulatory Activity. Bioinformatics.

[95] Gruber AR, Fallmann J, Kratochvill F, Kovarik P, Hofacker IL (2011). AREsite: a database for the comprehensive investigation of AU-rich elements. Nucleic Acids Res.

[96] Chou C-H (2016). miRTarBase 2016: updates to the experimentally validated miRNA-target interactions database. Nucleic Acids Res.

[97] Xiao F (2009). miRecords: an integrated resource for microRNA-target interactions. Nucleic Acids Res.

[98] Friedman J, Hastie T, Tibshirani R (2010). Regularization Paths for Generalized Linear Models via Coordinate Descent. J Stat Softw.

[99] Benjamini Y, Hochberg Y (1995). Controlling the False Discovery Rate: A Practical and Powerful Approach to Multiple Testing. Journal of the Royal Statistical Society. Series B (Methodological).

[100] McCarthy DJ, Smyth GK (2009). Testing significance relative to a fold-change threshold is a TREAT. Bioinformatics.

[101] Yates A (2016). Ensembl 2016. Nucleic Acids Res.

[102] Hubley R (2016). The Dfam database of repetitive DNA families. Nucleic Acids Res.

[103] Benson G (1999). Tandem repeats finder: a program to analyze DNA sequences. Nucleic Acids Res.

[104] Hecker N, Wiegels T, Torda AE (2013). RNA secondary structure diagrams for very large molecules: RNAfdl. Bioinformatics.

