# Supplementary material for: Associating transcription factors and conserved RNA structures with gene regulation in the human brain
Source: Sci Rep. 2017 Jul 18;7:5776. doi: 10.1038/s41598-017-06200-4 (PMC5516038; doi:10.1038/s41598-017-06200-4)
Supplement: Supplementary file 1 — S1 Supplementary text [file 41598_2017_6200_MOESM1_ESM.pdf]

Supplementary text - Associating transcription factors and conserved RNA structures with gene regulation in the human brain

Nikolai Hecker[1,2,3,4], Stefan E. Seemann[1,2], Asli Silahtaroglu[1,5], Walter L. Ruzzo[1,6,7,*] and Jan Gorodkin[1,2,*]

1 Center for non-coding RNA in Technology and Health
2 Department of Veterinary Clinical and Animal Sciences, University of Copenhagen, 1870 Frederiksberg C, Denmark
3 Max-Planck Institute of Molecular Cell Biology and Genetics, Pfoenhauerstr. 108, 01307 Dresden, Germany (current address)
4 Max-Planck Institute for the Physics of Complex Systems, Nöthnitzer Str. 38, 01187 Dresden, Germany (current address)
5 Department of Cellular and Molecular Medicine, University of Copenhagen, 2200 Copenhagen N, Denmark
6 Paul G. Allen School of Computer Science & Engineering, and Department of Genome Sciences, University of Washington, 185 Stevens Way, WA 98195-2350, Seattle, USA
7 Fred Hutchinson Cancer Research Center, 1100 Fairview Ave. N., WA 98109, Seattle, USA
∗ E-mail: gorodkin@rth.dk and ruzzo@cs.washington.edu

Brain region nomenclature

region ID	depth	acronym	name
4006	1	GM	Grey Matter
4007	2	Tel	Telencephalon
4391	2	DiE	Diencephalon
4833	2	MET	Metencephalon
9001	2	MES	Mesencephalon
9512	2	MY	Myelencephalon
4008	3	Cx	Cerebral Cortex
4275	3	CxN	Cerebral Nuclei
4392	3	TH	Thalamus
4540	3	Hy	Hypothalamus
4696	3	Cb	Cerebellum
9131	3	Pons	Pons
9002	3	MTg	Midbrain Tegmentum
4009	4	FL	Frontal Lobe
4084	4	PL	Parietal Lobe
4132	4	TL	Temporal Lobe
4180	4	OL	Occipital Lobe
4219	4	LL	Limbic Lobe
4300	4	BF	Basal Forebrain
4327	4	Amg	Amygdala
4276	4	BG	Basal Ganglia
4393	4	DT	Dorsal Thalamus
4665	4	MaR	Mammillary Region
4697	4	CbX	Cerebellar Cortex
9135	4	PTg	Pontine Tegmentum
4085	5	PoG	postcentral gyrus
4103	5	IPL	inferior parietal lobule
4220	5	CgG	Cingulate gyrus
4249	5	HiF	hippocampal formation
12930	5	CbH	Cerebellar Hemispheres
12931	6	Cb-AL	Anterior Lobe
12936	6	Cb-PL	Posterior Lobe

Table S1: Brain region abbreviations. We used the nomenclature and IDs defined by the Allen Brain Atlas. Except, we renamed some of the acronyms for illustrations. The brain hierarchy 'depth' was defined by a depth-first search starting from the entire brain.

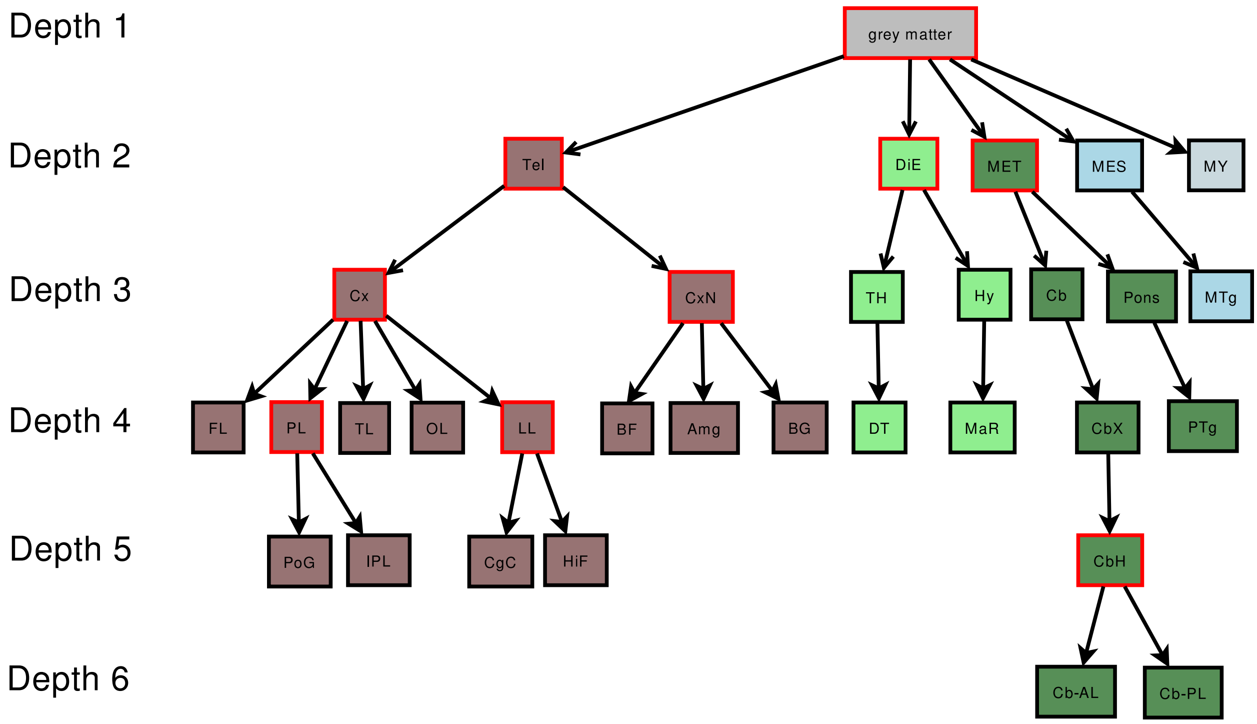

Figure S1: Brain region ontology. Black arrows show the sub regions that form a region at a more coarse-grained level of region separation. Red rectangles indicate that the region was considered for the differential expression analysis, i.e. the differential expression between the sub regions of the region was evaluated. Brain regions are defined as annotated in the Allen Human Brain Atlas. The selected regions contain at least 20 samples after collapsing replicates as we required for the transcription factor analysis. Abbreviations are explained in Table S1.

Micro array data set

The following section contains a brief outline about the data sets that were used for the analysis. For a detailed description about the micro arrays including normalization and sample preparation refer to the Allen Brain Atlas documentation *Microarray Survey*, *Microarray Data Normalization* and *Case Qualification and Donor Profiles* at:

http://help.brain-map.org/display/humanbrain/Documentation

The following description of the experimental setup of is a condensed summary of the documents mentioned above. The Allen Atlas Brain Atlas contains micro-array data derived from region samples that represents all anatomical structures throughout the human brain (Table S2) [13]. These samples were obtained from post-mortem tissue of six human donors through manual macro-dissection and laser micro-dissection (LMD). We focused on micro array data because RNA-seq data is only available for two of the six donors. Hence, the RNA-seq data would provide less robust statistics. The six human brain donors have no known neuropsychiatric or neuropathological history and blood samples were investigated for the presence of substances that could point to neuropathology or neuropsychiatric disorders including substance abuse addictions through toxicology screening. Gross neuropathology of the human brains was assessed through magnetic resonance imaging (MRI) or by pathologist during initial dissection and procession. Micro-neuropathology was assessed through different histological methods including thionin-based Nissl-staining and silver-staining.

ID	AID	FID	sex	ethnicity	age	PMI	handedness	LB	RB
1	H0351.2002	10021	male	African American	39yr	10h	left	true	true
2	H0351.1009	12876	male	Caucasian	57yr	25.5h	cross-dominant	true	false
3	H0351.1012	14380	male	Caucasian	31yr	17.5h	right	true	false
4	H0351.1015	15496	female	Hispanic	49yr	30h	right	true	false
5	H0351.1016	15697	male	Caucasian	55yr	18h	right	true	false
6	H0351.2001	9861	male	African American	24yr	23h	left	true	true

Table S2: Information about human donors. 'ID' refers to the identifier that was assigned to the human donor inside the Allen Brain Atlas. 'PMI' refers to the post-mortem interval, i.e. the approximate number of hours passed after the death of the donor before the samples were obtained. 'LB' and 'RB' indicate that the samples were taken from the left or right brain hemisphere, respectively.

Frozen brain regions were first partitioned into larger slabs. Samples from the cerebral cortex, cerebellar cortex and the brain stem were obtained through manual macro-dissection corresponding to anatomical brain regions. Samples for sub-cortical regions and cerebellar nuclei were obtained through LMD and subjected to a MELT enzyme cocktail (Ambion) prepared with beta-mercaptoethanol (BME). A DNase step was performed for removing DNA. Instead of the MELT protocol, RNA from macro-dissected samples was isolated using a guanidium-thiocyanate-phenol-chloroform based extraction method (TRI Reagent, Applied Biosystems). Lysate corresponding to macro-dissected samples were homogenized using an Omni-Prep Homogenizer. DNA was removed through phase separation by centrifugation after the lysates were added to a bromochloropropan (BCP) solution. Reagents from a custom Ambion RNA isolation kit were used for RNA isolation for macro-dissected samples. The RNA for all samples was then isolated using the magnetic bead-system MagMAX Express 96 (Applied Biosystems). RNA quality for all samples was assessed using a Bioanalyzer (Agilent Technologies) with RNA samples loaded onto a Pico Chip requiring an RNA integrity number (RIN) of at least 5.5 in general [11], no significant contamination by rRNA degradation or other RNA degradation products and the presence of no noticeable DNA or background contaminants, as well as a sufficient quantity of RNA.

Micro array experiments were performed by Coulter Genomics for three brains and by Covance Genomics for the other three brains. Micro array data was processed for array specific biases including the GC-content of the probes, location on the chip and experiment-wise mean intensities. Next, a 75%-quartile alignment of the micro array intensities was performed within each batch inside the same brain. This was followed by an adjustment of RNA quality differences among samples within a batch using a local regression model based on the RIN values for the samples. This was followed by an adjustment for batch effects within the same brain. Here, mean expression values were aligned to control samples that were present in each batch and a quantile-quantile normalization was performed that considers differences between samples derived from macro-dissection and LMD. To address differences in expression values between the different brains, expression values of control samples were aligned between all batches of all brains and the mean values for the expression inside each brain was aligned between the brains.

We downloaded the $\log_2$-normalized micro array data of the six human donors from the Allen Brain Atlas:

http://human.brain-map.org/static/download

The micro arrays contain an extended 4x44K Agilent Whole Human Genome probe set. For the analysis, we only considered probes that were annotated with EntrezGeneIDs. In total, these probes cover 20,787 genes. For the majority of genes, there are two or more different probes that correspond to different exons when possible. Since the samples were only obtained from the left brain hemisphere for the majority of donors we only used samples from left brain hemispheres to avoid bias introduced by the different donors. Figure S1 shows the hierarchy of the analyzed brain regions. For each, region we combined all samples of its sub regions from all of the six donors. This means a region at a lower brain hierarchy depth is a superset of the samples of its sub regions. For the gene transcription factor interaction regression analysis, we collapsed probes that correspond to the same EntrezGeneID by the median expression value and then replicated samples of the

same donor by the median expression value. For the differential expression analysis, we did not collapse any samples or any replicates. Table S3 summarizes the number of samples before and after collapsing replicates.

					all samples of each donor						collapsed samples of each donor					
depth	region ID	Abr	n_{all}	n_{Call}	n_1	n_2	n_3	n_4	n_5	n_6	n_{C1}	n_{C2}	n_{C3}	n_{C4}	n_{C5}	n_{C6}
2	4007	Tel	1731	556	256	236	335	280	307	317	91	89	97	94	99	86
2	4391	DiE	252	155	38	47	52	38	43	34	21	29	31	27	30	17
2	4833	MET	454	222	77	48	74	91	106	58	36	24	30	42	48	42
2	9001	MES	135	66	33	9	22	24	18	29	15	6	13	10	10	12
2	9512	My	203	72	46	16	44	35	23	39	14	8	12	13	11	14
3	4008	Cx	1452	446	206	196	287	241	260	262	75	69	78	75	79	70
3	4275	CxN	279	110	50	40	48	39	47	55	16	20	19	19	20	16
3	4392	TH	145	64	24	26	28	21	21	25	9	11	11	11	11	11
3	4540	Hy	87	78	11	17	20	16	18	5	10	15	17	15	17	4
3	4696	Cb	316	160	50	42	48	62	80	34	23	21	20	31	35	30
3	9131	Pons	138	62	27	6	26	29	26	24	13	3	10	11	13	12
3	9002	MTg	118	55	29	8	21	20	14	26	12	5	12	8	8	10
4	4009	FL	394	129	73	49	71	64	61	76	24	18	23	21	23	20
4	4084	PL	217	72	26	37	42	32	41	39	12	12	12	11	13	12
4	4132	TL	342	93	40	37	73	51	66	75	15	15	16	13	17	17
4	4180	OL	175	60	23	25	38	35	35	19	10	10	10	13	10	7
4	4219	LL	291	80	41	44	55	52	51	48	12	12	15	15	14	12
4	4276	BG	149	41	30	20	26	21	23	29	7	7	6	7	7	7
4	4300	BF	43	27	5	7	9	6	12	4	2	6	6	5	6	2
4	4327	Amg	54	36	9	8	6	7	10	14	6	6	6	6	6	6
4	4393	DT	120	52	19	21	23	16	19	22	7	9	9	9	9	9
4	4665	MaR	32	29	3	6	7	7	6	3	3	6	6	6	6	2
4	4697	CbX	295	147	48	40	44	60	76	27	22	20	17	30	32	26
4	9135	PTg	103	56	21	2	19	21	21	19	12	2	9	10	12	11
5	4085	PoG	62	22	12	13	10	5	10	12	4	4	4	3	3	4
5	4103	IPL	68	24	7	10	13	12	11	15	4	4	4	4	4	4
5	4220	CgC	112	29	11	23	23	25	21	9	4	4	6	6	5	4
5	4249	HiF	130	36	26	14	22	17	20	31	6	6	6	6	6	6
6	12931	Cb-AL	49	25	2	12	12	8	12	3	1	6	5	4	6	3
6	12936	Cb-PL	158	73	24	28	31	25	38	12	10	14	11	13	13	12

Table S3: Number of samples per region. The table enumerates the number of samples for each region that was used in the analysis. The regions associated with a given 'region ID' and 'Abr' are listed in Table S1. n_{all} refers to the number of samples for a region combined from all six donors. $n_1, n_2, n_3, n_4, n_5, n_6$ are the number of samples for each donor for the indicated region. The numbers refer to the donor IDs as specified in Table S2. Similarly, n_{Call} refers to the number of samples for a region combined from all six donors after collapsing replicated samples from the same donor. $n_{C1}, n_{C2}, n_{C3}, n_{C4}, n_{C5}, n_{C6}$ are the number of samples for each donor for the indicated region after collapsing replicated samples from the same donor.

Statistical tests

ID	depth	abbreviation	region	contrasts
4006	2	GM	grey matter	Tel↔DiE, Tel↔MET, Tel↔MES, Tel↔My, DiE↔MET, DiE↔MES, DiE↔My, MET↔MES, MET↔My, MES↔My
4007	3	Tel	telencephalon	Cx↔CxN
4391	3	DiE	diencephalon	TH↔Hy
4833	3	MET	metencephalon	Cb↔Pons
4008	4	Cx	cerebral cortex	FL↔PL, FL↔TL, FL↔OL, FL↔LL, PL↔TL, PL↔OL, PL↔LL, TL↔OL, TL↔LL, OL↔LL
4275	4	CxN	cerebral nuclei	BF↔Amg, BF↔BG, Amg↔BG
4084	5	PL	parietal lobe	PoG↔IPL
4219	5	LL	limbic lobe	CgC↔HiF
12930	6	CbH	cerebellar hemispheres	Cb-AL↔Cb-PL

Table S4: Specified contrasts for the differential expression analysis. For each region, pair-wise combinations of its sub regions were analyzed for differential expression analysis. 'contrasts' refers to the specified contrasts for the differential expression analysis. Here, A↔B indicates a comparison between the samples of region A and region B. A and B refer to region abbreviations as specified in Table S1. 'ID' is the corresponding region ID. The 'depth' indicates the level in the brain ontology hierarchy as illustrated by figure S1.

(A) RE enrichment

	diff. expr.	not diff. expr.
RE	$x_{1,1}$	$x_{1,2}$
no RE	$x_{2,1}$	$x_{2,2}$

(B) structured RE enrichment

	diff. expr.	not diff. expr.
structured RE	$x_{1,1}$	$x_{1,2}$
no structured RE	$x_{2,1}$	$x_{2,2}$

Figure S2: Contingency tables for the RE enrichment analysis. To assess whether there is an enrichment between regulatory elements (RE) and differently expressed genes ('diff expr.'), χ^2-test were performed based on the depicted 'contingency tables' where $x_{1,1}, x_{2,1}, x_{2,1}, x_{2,2}$ correspond to the number of genes that satisfy the criteria specified in the column and row. For instance, $x_{1,1}$ refers to the number of genes that were differently expressed between the analyzed sub regions and that contain at least one RE of specific type, e.g. a AU-rich element. Here, 'structured RE' refers to REs that also overlap predictions for conserved RNA secondary structures. The null hypothesis for the χ^2-test is that the log-odds-ratio $\log(\frac{x_{1,1}}{x_{1,2}}) - \log(\frac{x_{2,1}}{x_{2,2}})$ does not differ significantly from zero, i.e. in our scenario that the presence of REs is independent of the differential expression of genes. If the null hypothesis is rejected, a plausible explanation would be a potential association between the presence of REs and differentially expressed genes.

Region specific transcription factor contributions

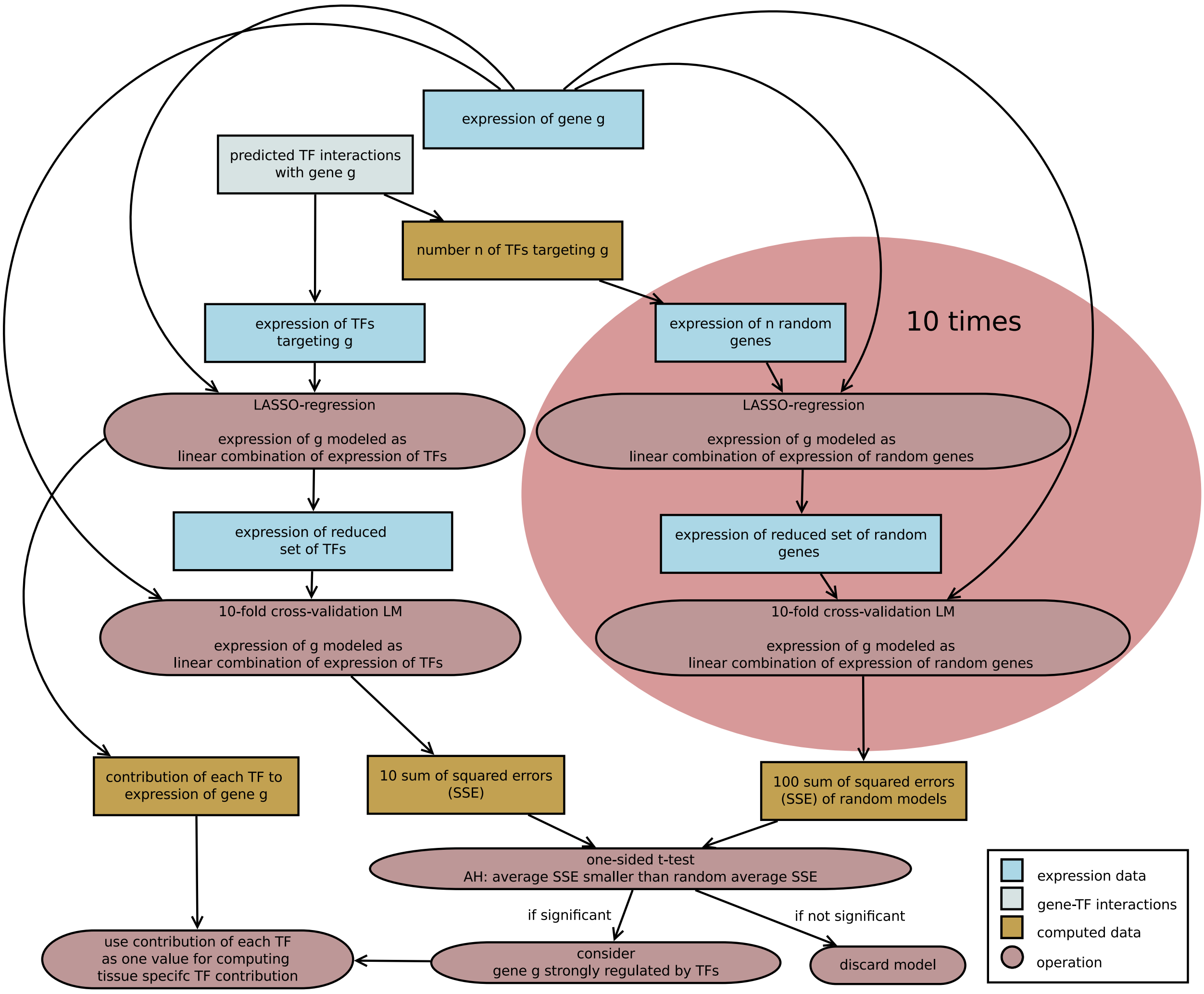

Figure S3: Flowchart for the transcription factor regression analysis. It is depicted how the expression of a gene g is modeled by the expression of transcription factors (TF) and how the significance that a resulting model is better than a random model was assessed. 'LM' refers to linear models that were generated with the R function 'lm'. The '10 times' indicates that the steps inside the red bubble were repeat ten times. 'AH' refers to the alternative statiscal hypothesis for the t-test. The null hypothesis is that the sum of squared errors are the same for the real and random models.

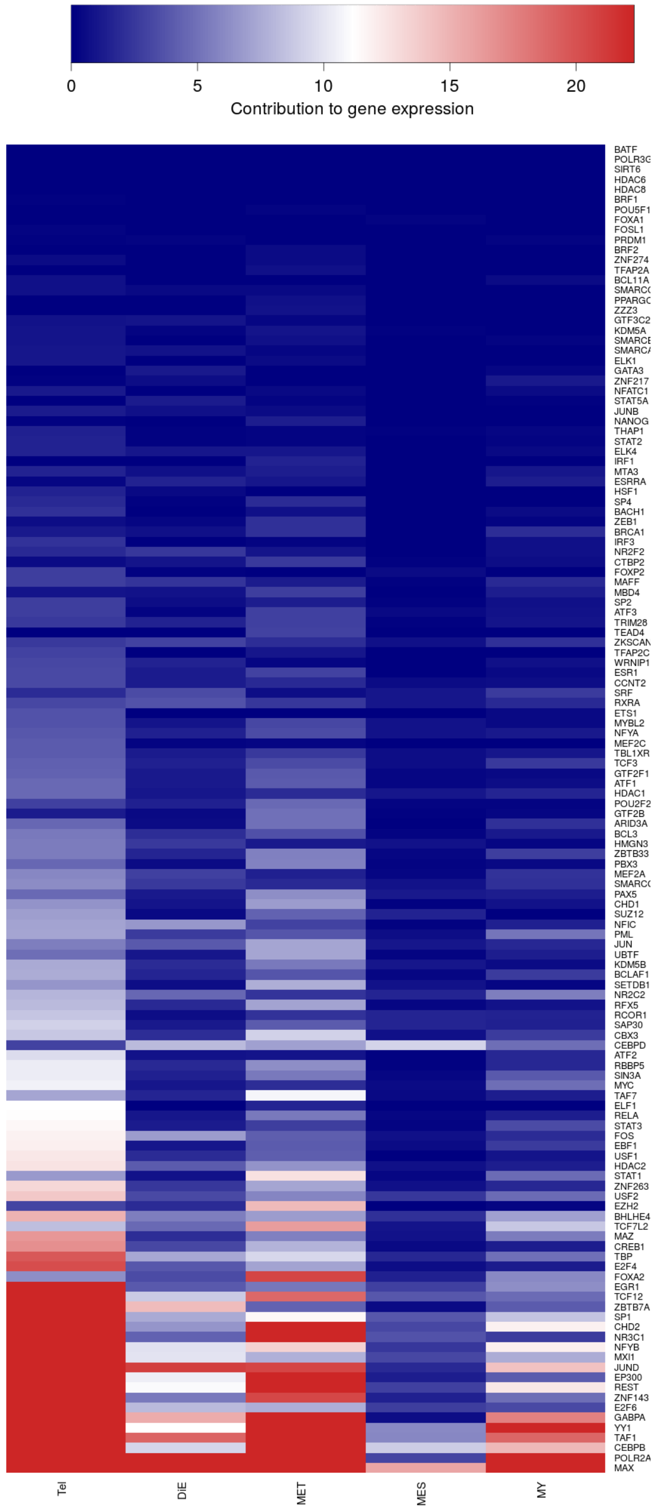

Figure S4: Transcription factor contribution to gene expression at depth 2 for run 1. The color gradient indicates the contribution to gene expression and is capped between 5% and 95% quantiles for all transcription factors (rows) in all depicted regions (columns). Abbreviations are specified inside Table S1.

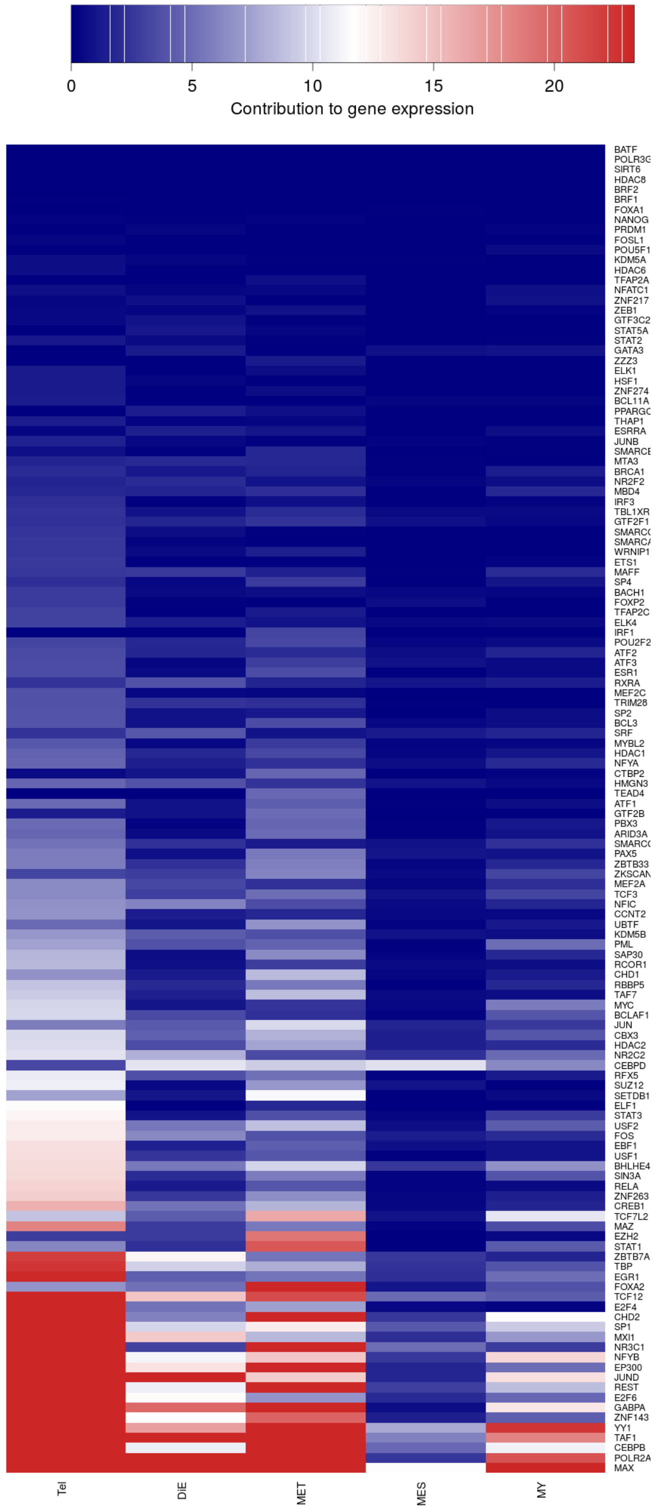

Figure S5: Transcription factor contribution to gene expression at depth 2 for run 2. The color gradient indicates the contribution to gene expression and is capped between 5% and 95% quantiles for all transcription factors (rows) in all depicted regions (columns). Abbreviations are specified inside Table S1.

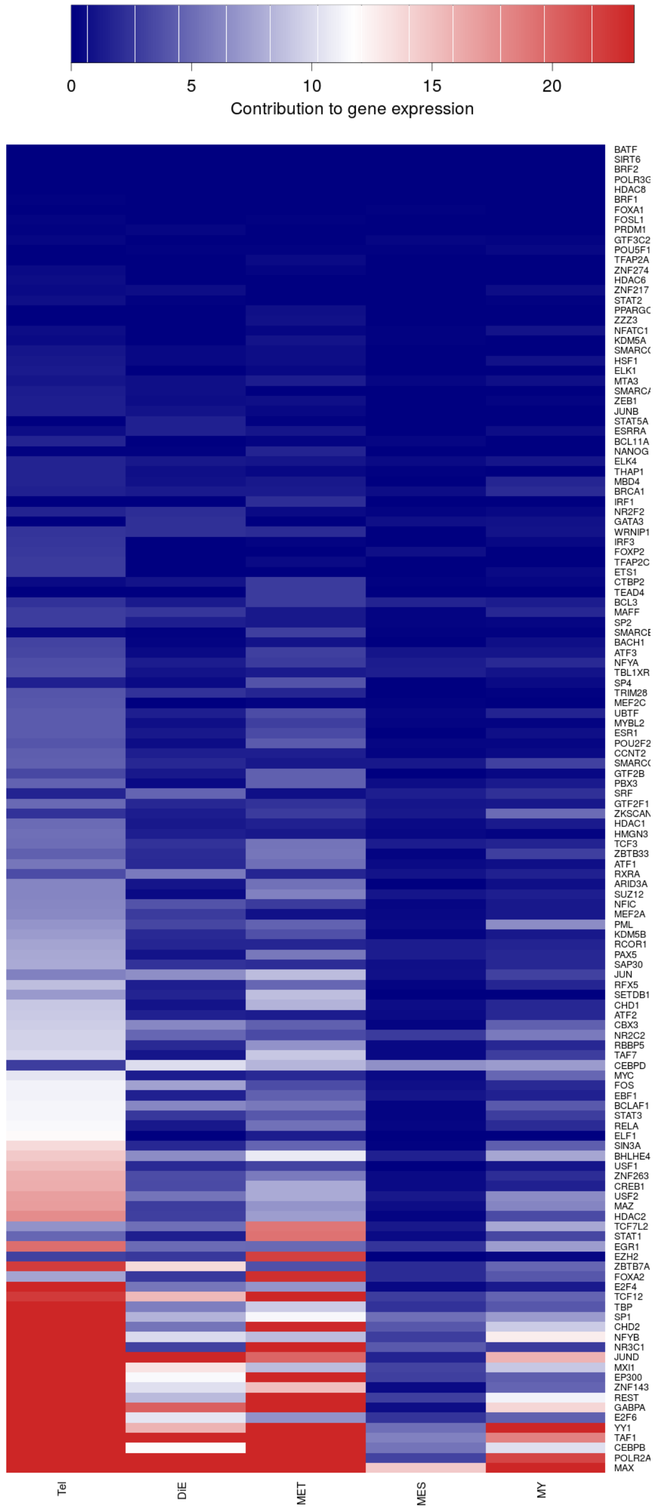

Figure S6: Transcription factor contribution to gene expression at depth 2 for run 3. The color gradient indicates the contribution to gene expression and is capped between 5% and 95% quantiles for all transcription factors (rows) in all depicted regions (columns). Abbreviations are specified inside Table S1..

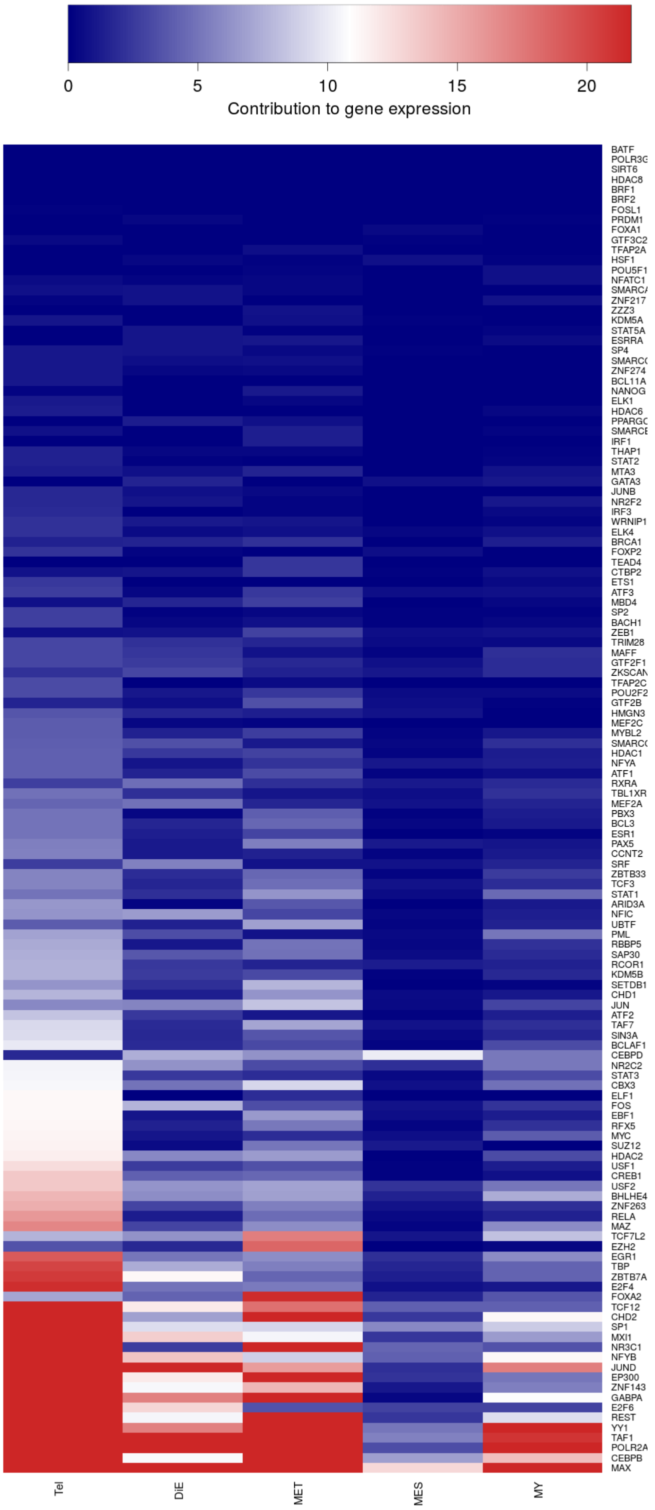

Figure S7: Transcription factor contribution to gene expression at depth 2 for run 4. The color gradient indicates the contribution to gene expression and is capped between 5% and 95% quantiles for all transcription factors (rows) in all depicted regions (columns). Abbreviations are specified inside Table S1.

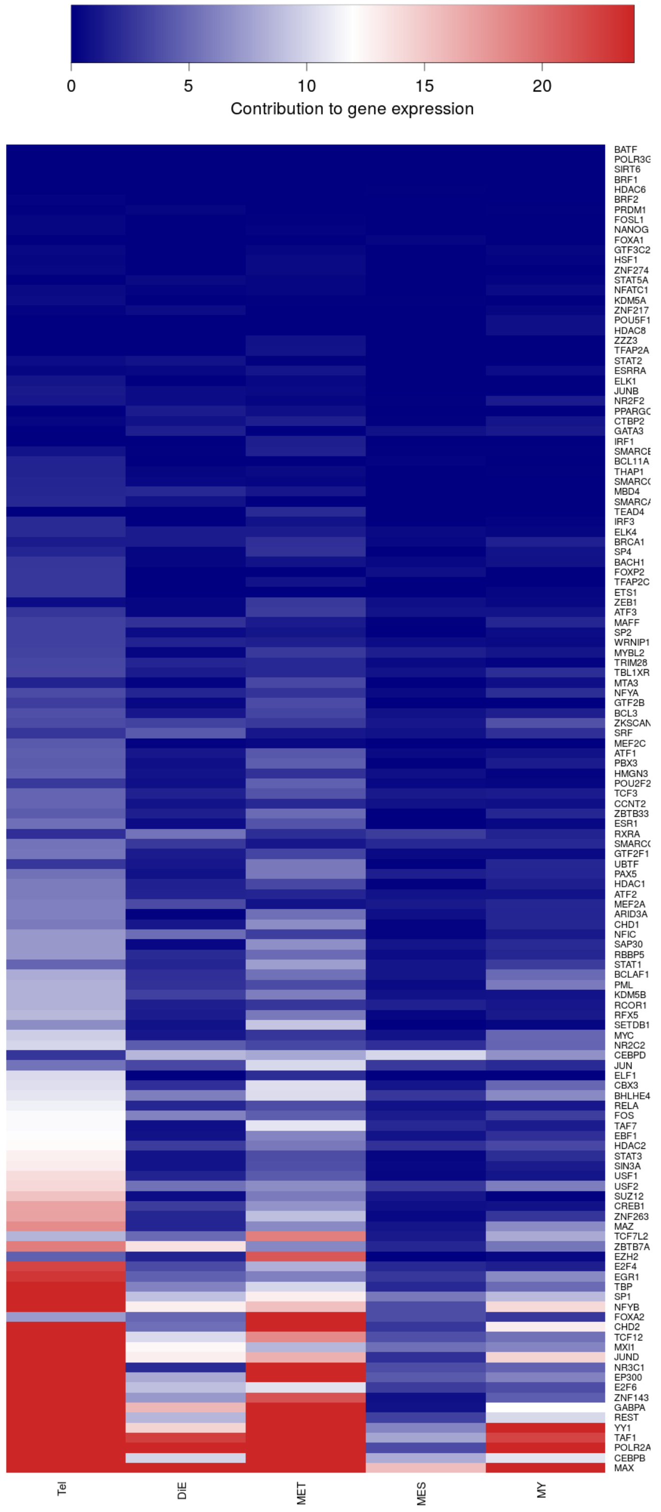

Figure S8: Transcription factor contribution to gene expression at depth 2 for run 5. The color gradient indicates the contribution to gene expression and is capped between 5% and 95% quantiles for all transcription factors (rows) in all depicted regions (columns). Abbreviations are specified inside Table S1.

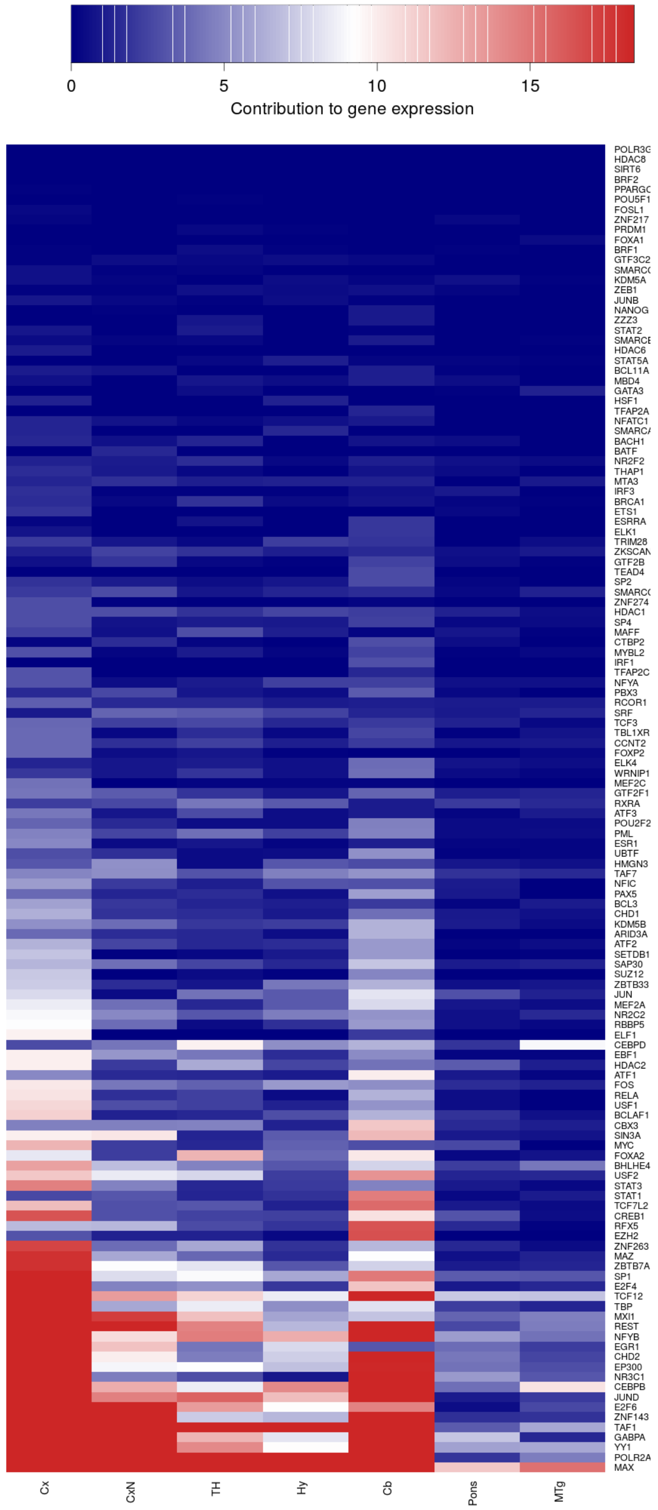

Figure S9: Transcription factor contribution to gene expression at depth 3 for run 1. The color gradient indicates the contribution to gene expression and is capped between 5% and 95% quantiles for all transcription factors (rows) in all depicted regions (columns). Abbreviations are specified inside Table S1.

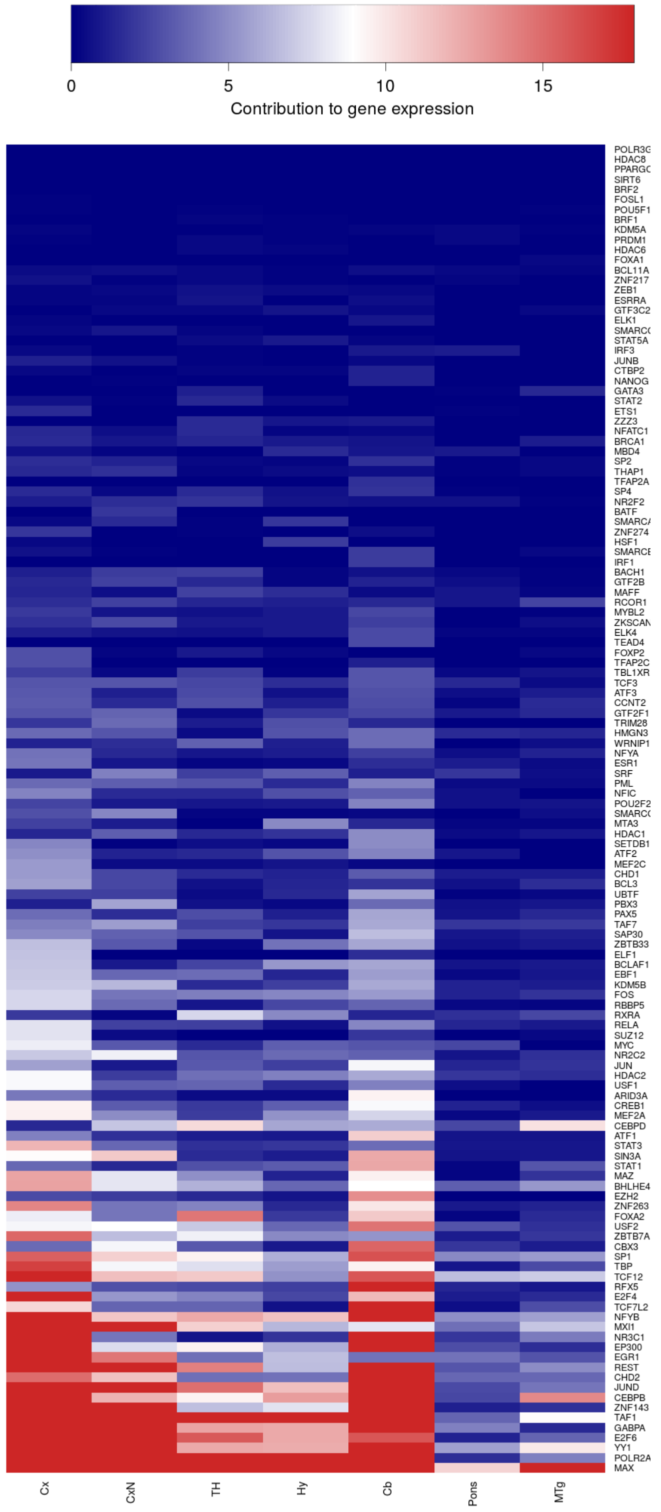

Figure S10: Transcription factor contribution to gene expression at depth 3 for run 2. The color gradient indicates the contribution to gene expression and is capped between 5% and 95% quantiles for all transcription factors (rows) in all depicted regions (columns). Abbreviations are specified inside Table S1.

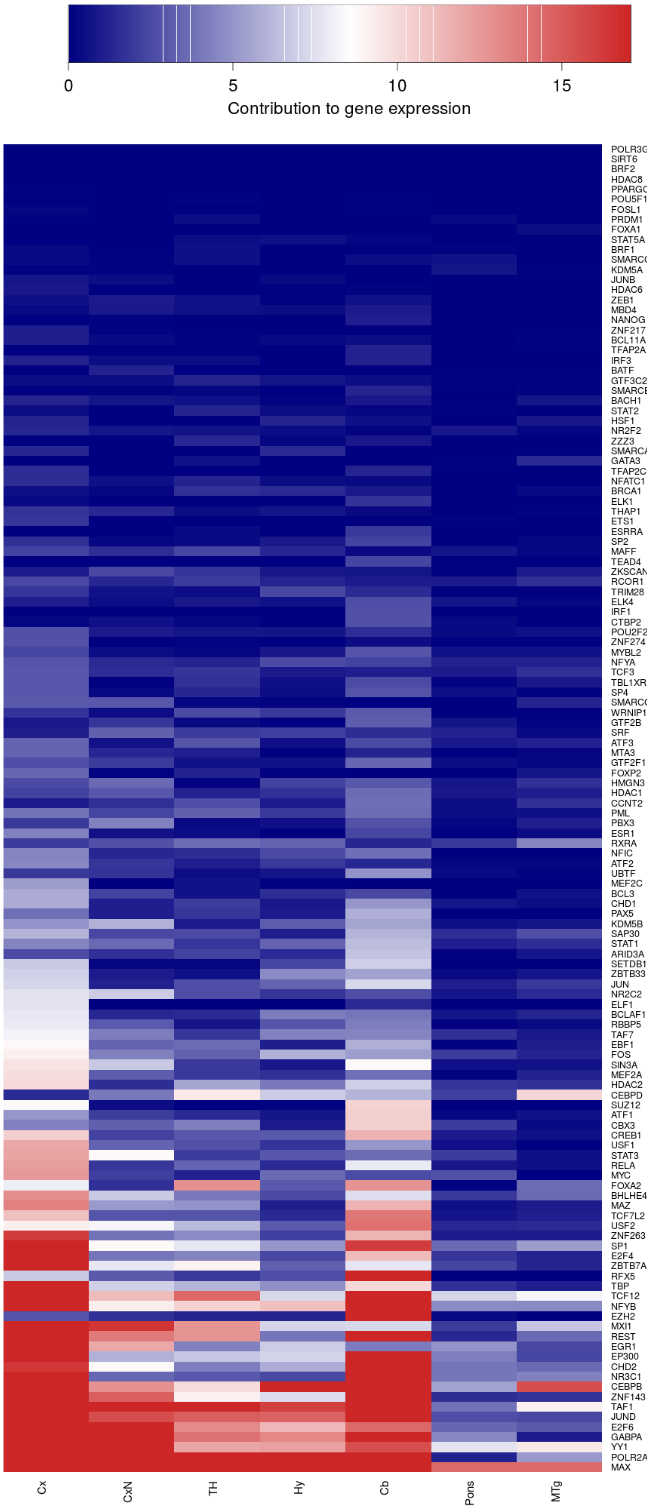

Figure S11: Transcription factor contribution to gene expression at depth 3 for run 3. The color gradient indicates the contribution to gene expression and is capped between 5% and 95% quantiles for all transcription factors (rows) in all depicted regions (columns). Abbreviations are specified inside Table S1.

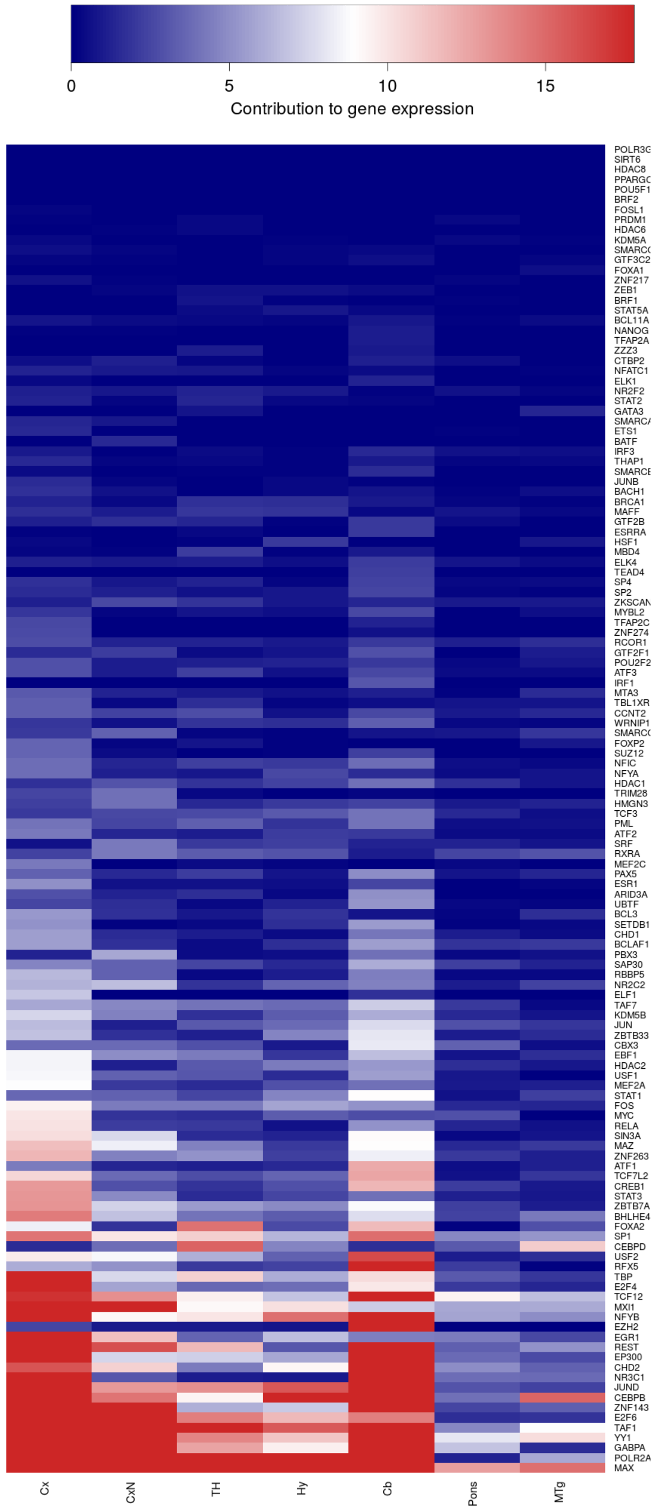

Figure S12: Transcription factor contribution to gene expression at depth 3 for run 4. The color gradient indicates the contribution to gene expression and is capped between 5% and 95% quantiles for all transcription factors (rows) in all depicted regions (columns). Abbreviations are specified inside Table S1.

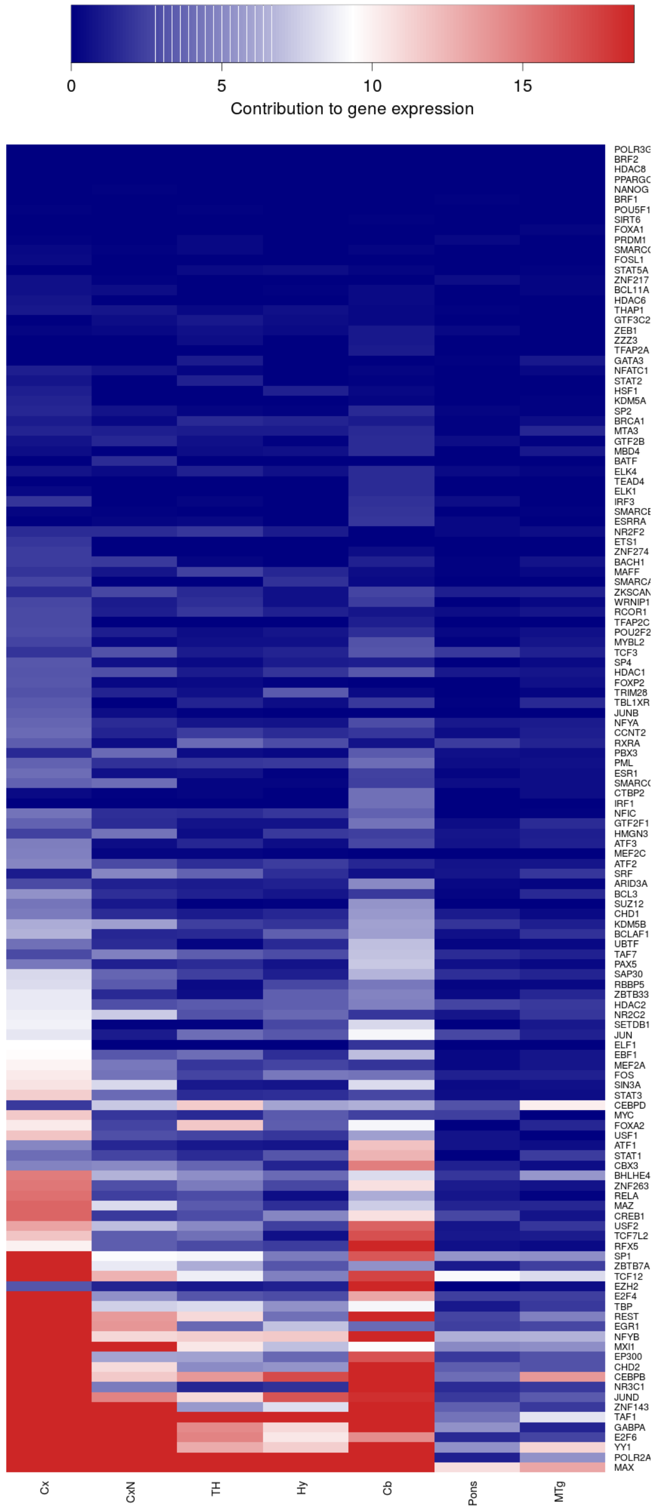

Figure S13: Transcription factor contribution to gene expression at depth 3 for run 5. The color gradient indicates the contribution to gene expression and is capped between 5% and 95% quantiles for all transcription factors (rows) in all depicted regions (columns). Abbreviations are specified inside Table S1.

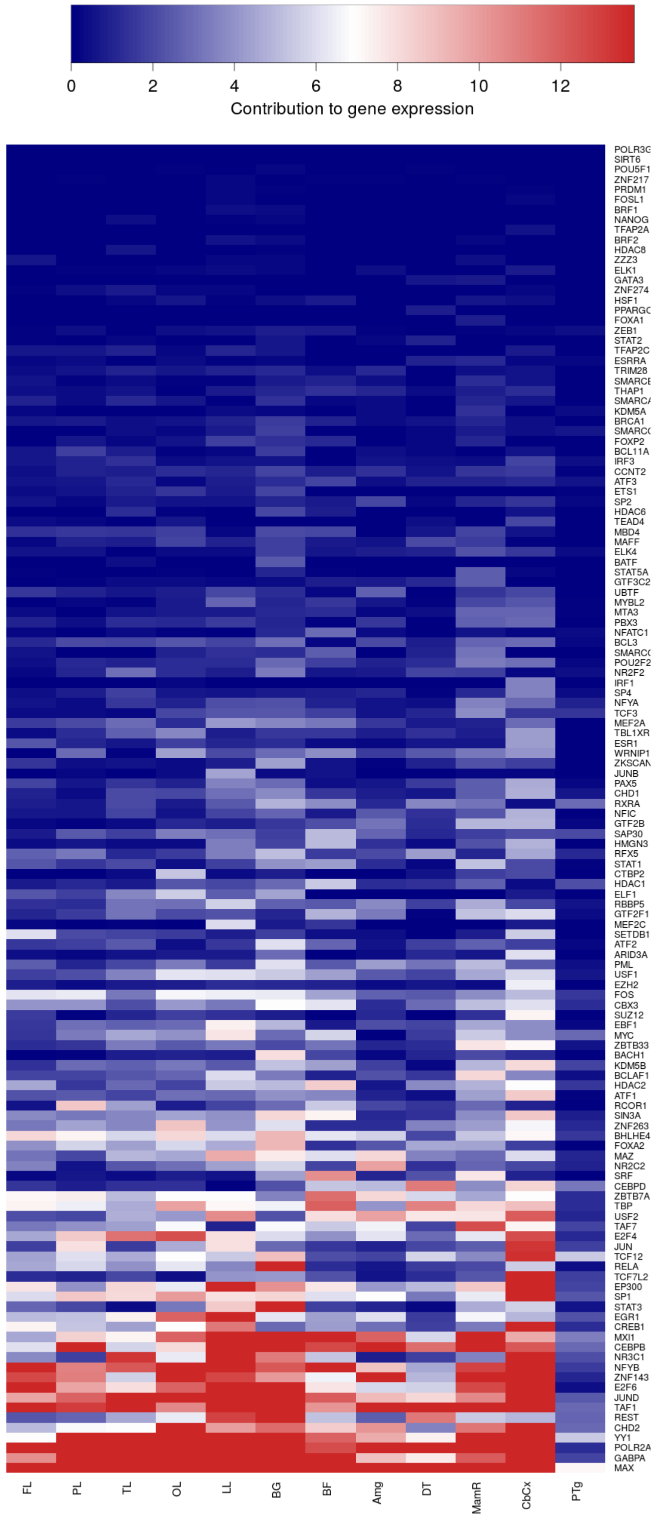

Figure S14: Transcription factor contribution to gene expression at depth 4 for run 1. The color gradient indicates the contribution to gene expression and is capped between 5% and 95% quantiles for all transcription factors (rows) in all depicted regions (columns). Abbreviations are specified inside Table S1.

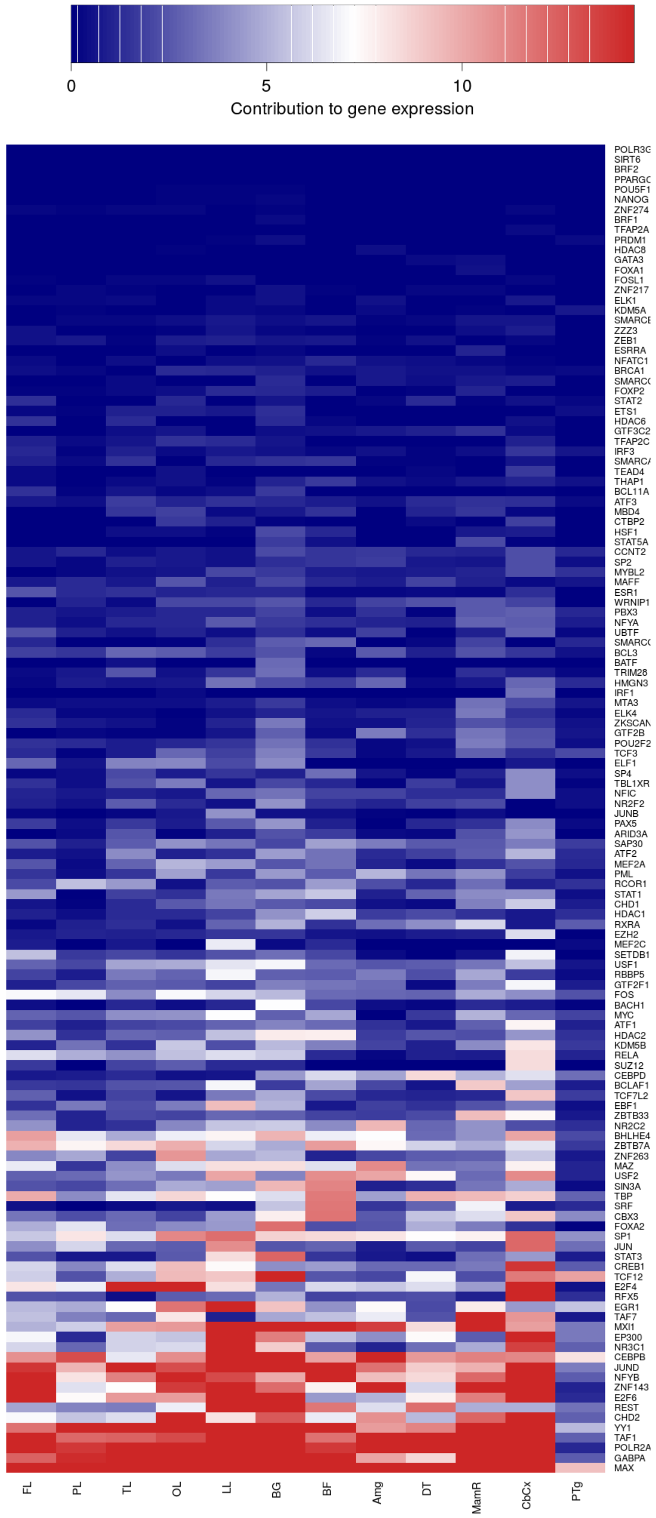

Figure S15: Transcription factor contribution to gene expression at depth 4 for run 2. The color gradient indicates the contribution to gene expression and is capped between 5% and 95% quantiles for all transcription factors (rows) in all depicted regions (columns). Abbreviations are specified inside Table S1.

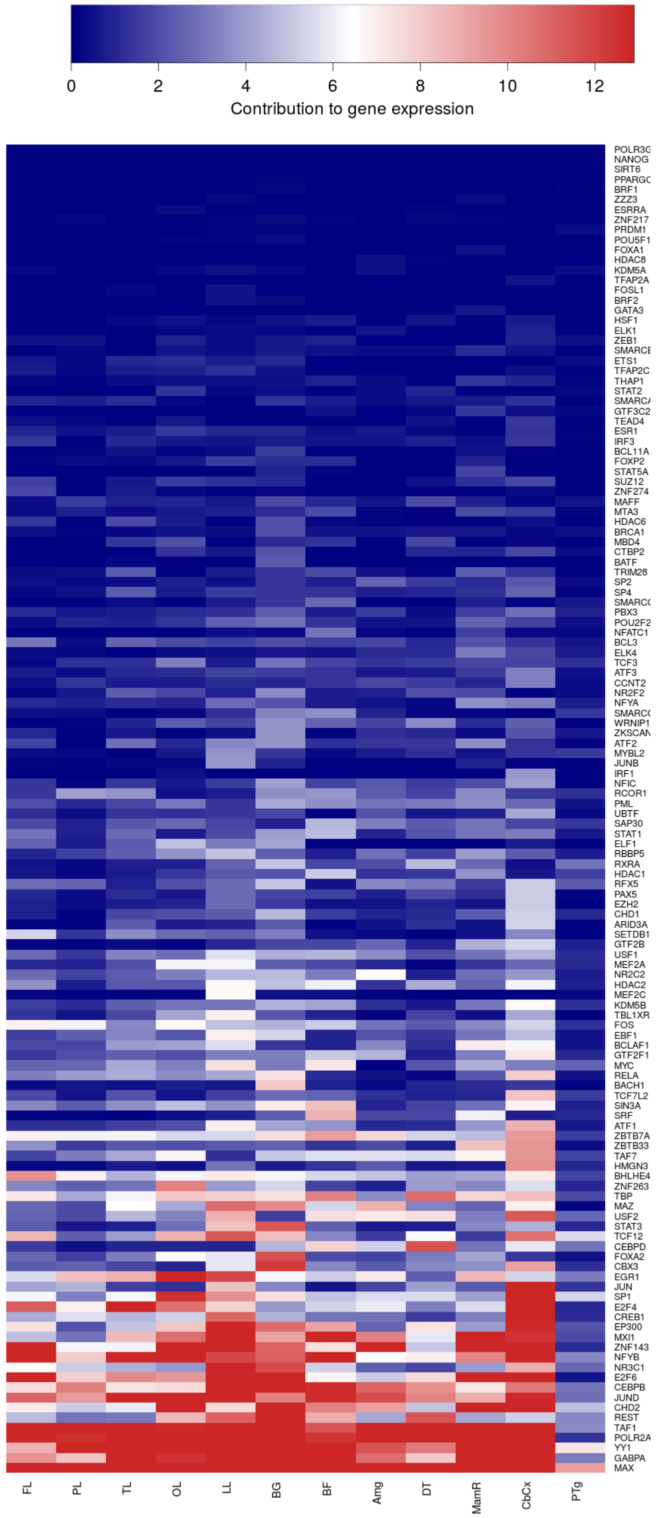

Figure S16: Transcription factor contribution to gene expression at depth 4 for run 3. The color gradient indicates the contribution to gene expression and is capped between 5% and 95% quantiles for all transcription factors (rows) in all depicted regions (columns). Abbreviations are specified inside Table S1.

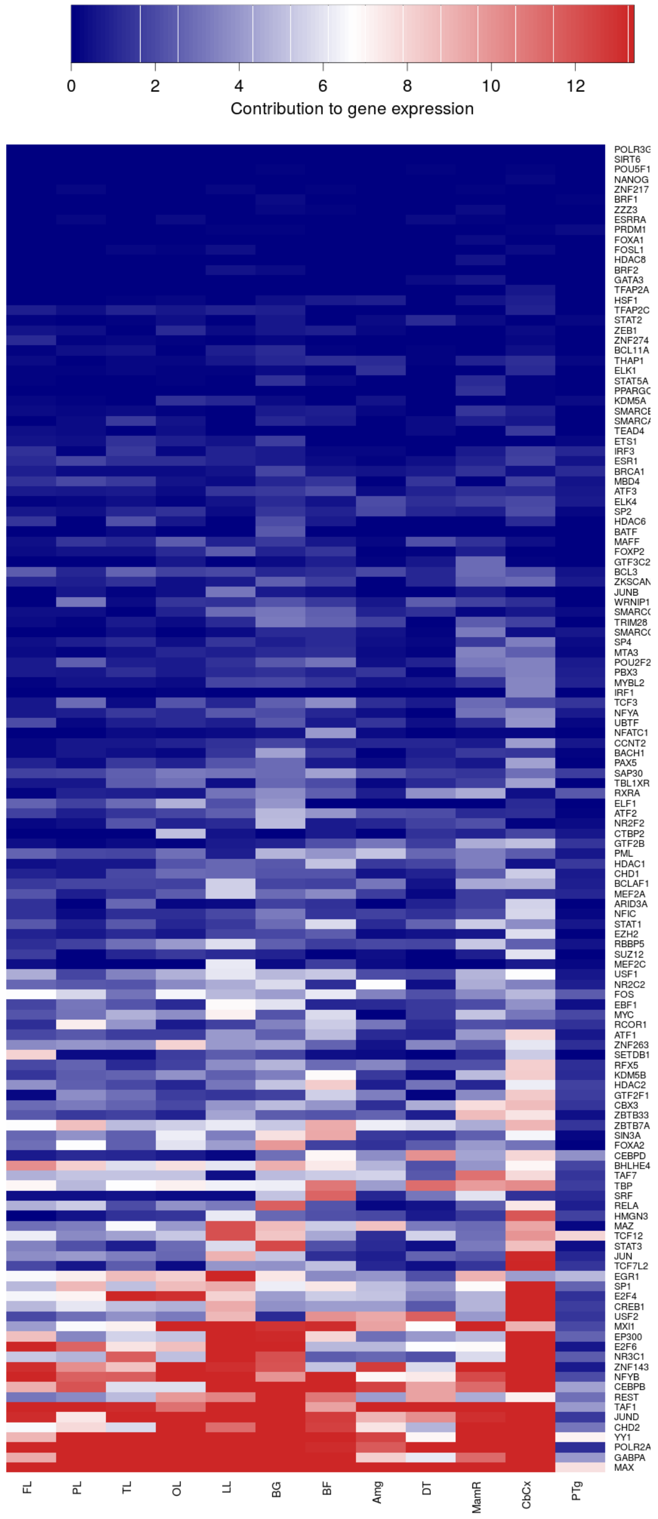

Figure S17: Transcription factor contribution to gene expression at depth 4 for run 4. The color gradient indicates the contribution to gene expression and is capped between 5% and 95% quantiles for all transcription factors (rows) in all depicted regions (columns). Abbreviations are specified inside Table S1.

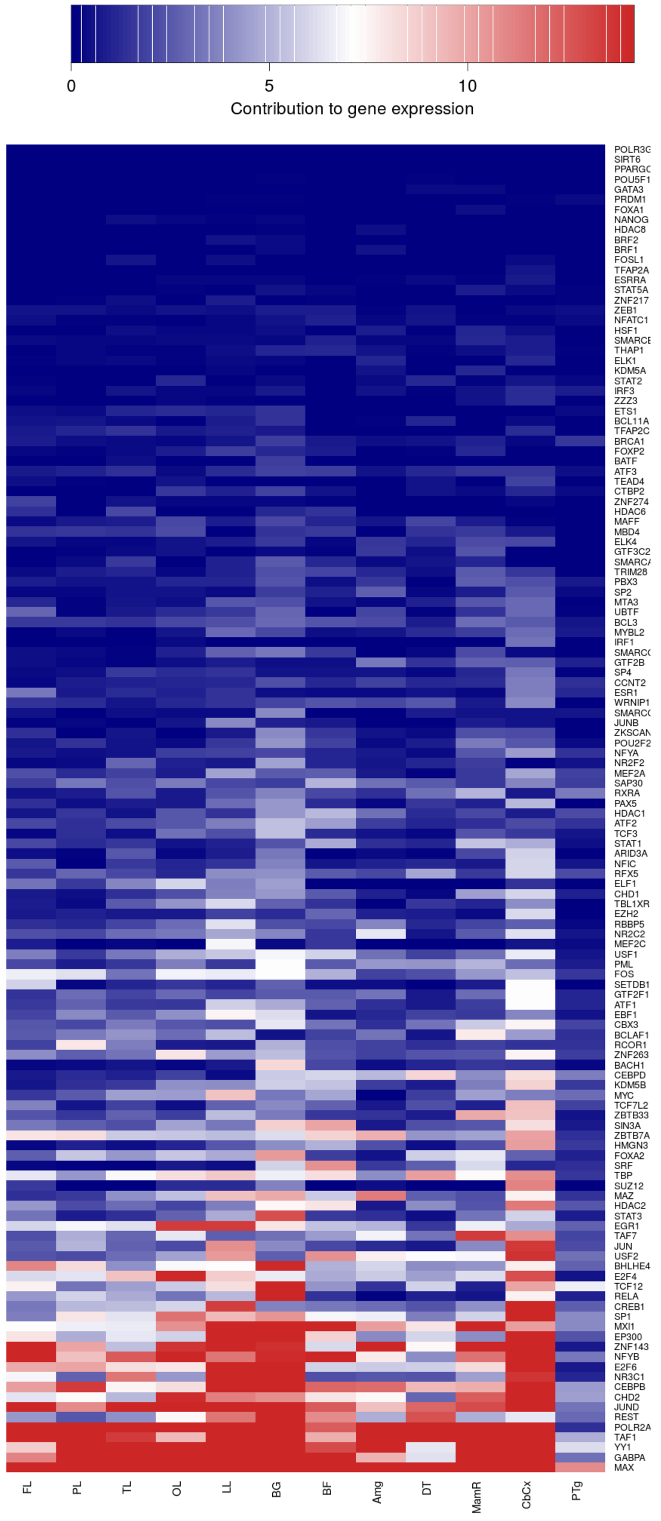

Figure S18: Transcription factor contribution to gene expression at depth 4 for run 5. The color gradient indicates the contribution to gene expression and is capped between 5% and 95% quantiles for all transcription factors (rows) in all depicted regions (columns). Abbreviations are specified inside Table S1.

Extended discussion of TF regulated genes

SRF showed a localized contribution to gene expression inside the Basal forebrain including the regulation of two F-box proteins FBXL16 and FXBO46. Among other functions, most F-box proteins facilitate substrate binding of ubiquitin ligases complexes, thus constitute important factors for protein degradation [9]. F box proteins such F box protein 7 and F-box protein 2 have been proposed to play crucial functions for the developing Parkinson's disease and Alzheimer's, respectively [3, 6]. Previous studies have suggested several functions for SRF inside the CNS including the morphological development of the cyto-architecture of neurons, migration of neurons to the olfactory bulp during development, myelinization of neurons, neurite growth, axon guidance and a link to antisocial behavior in adult mice [7, 12]. SRF is one of the targets for anti-depressive drug development [5]. While we did capture these functions for SRF in the basal forebrain based on our relatively restrictive model, we can speculate that abnormal expression of SRF in the Basal forebrain poses a potential risk factor for developing neurological disorders due to misregulation of the F-box proteins FBXL16 and FXBO46.

Enriched REs in inside differentially expressed mRNAs

(A) 5′ UTRs

region	depth	ARE	RBP	HCE	miRNA	APA	ALU
Grey matter	2	-	■ (both)	■ (RE)	-	-	-
Telencephalon	3	-	■ (structured RE)	■ (both)	-	-	-
Diencephalon	3	-	-	■ (RE)	-	-	-
Metencephalon	3	-	■ (both)	■ (RE)	■ (RE)	-	-
Cerebral Cortex	4	-	-	■ (RE)	-	-	-
Cerebral Nuclei	4	-	-	■ (RE)	-	-	-
Limbic Lobe	5	-	■ (both)	■ (RE)	■ (RE)	-	■ (RE)
Parietal Lobe	5	-	-	-	-	-	-
Cerebellar Hemispheres	6	-	-	-	-	-	-

■ (RE): RE ■ (structured RE): structured RE ■ (both): both

(B) 3′ UTR

region	depth	ARE	RBP	HCE	miRNA	APA	ALU
Grey matter	2	■ (both)	■ (both)	■ (structured RE)	■ (both)	■ (both)	■ (RE)
Telencephalon	3	■ (both)	■ (both)	■ (RE)	■ (both)	■ (RE)	-
Diencephalon	3	■ (both)	■ (both)	■ (structured RE)	■ (structured RE)	■ (both)	■ (structured RE)
Metencephalon	3	■ (both)	■ (both)	■ (both)	■ (both)	■ (both)	■ (RE)
Cerebral Cortex	4	■ (RE)	-	■ (RE)	-	-	-
Cerebral Nuclei	4	■ (both)	-	■ (both)	-	■ (RE)	■ (structured RE)
Limbic Lobe	5	■ (both)	■ (both)	■ (both)	■ (both)	■ (both)	-
Parietal Lobe	5	-	-	-	-	-	-
Cerebellar Hemispheres	6	-	-	-	-	-	-

■ (RE): RE ■ (structured RE): structured RE ■ (both): both

Figure S19: Enrichment for different types of regulatory elements inside either 5′ UTRs or 3′ UTRs. Depicted is the enrichment for differentially expressed genes inside the indicated region that contain at least one RE of the indicated type inside their UTRs. A) 5′ UTRs and B) 3′ UTRs were analyzed separately. We distinguished between the presence of a non-structured RE (red square), i.e. REs that do not overlap predicted RNA secondary structures and structured REs (blue square), i.e. REs that overlap predicted RNA secondary structures. Violet squares indicate an enrichment for both non-structured REs and structured REs.

Phylogenetic tree

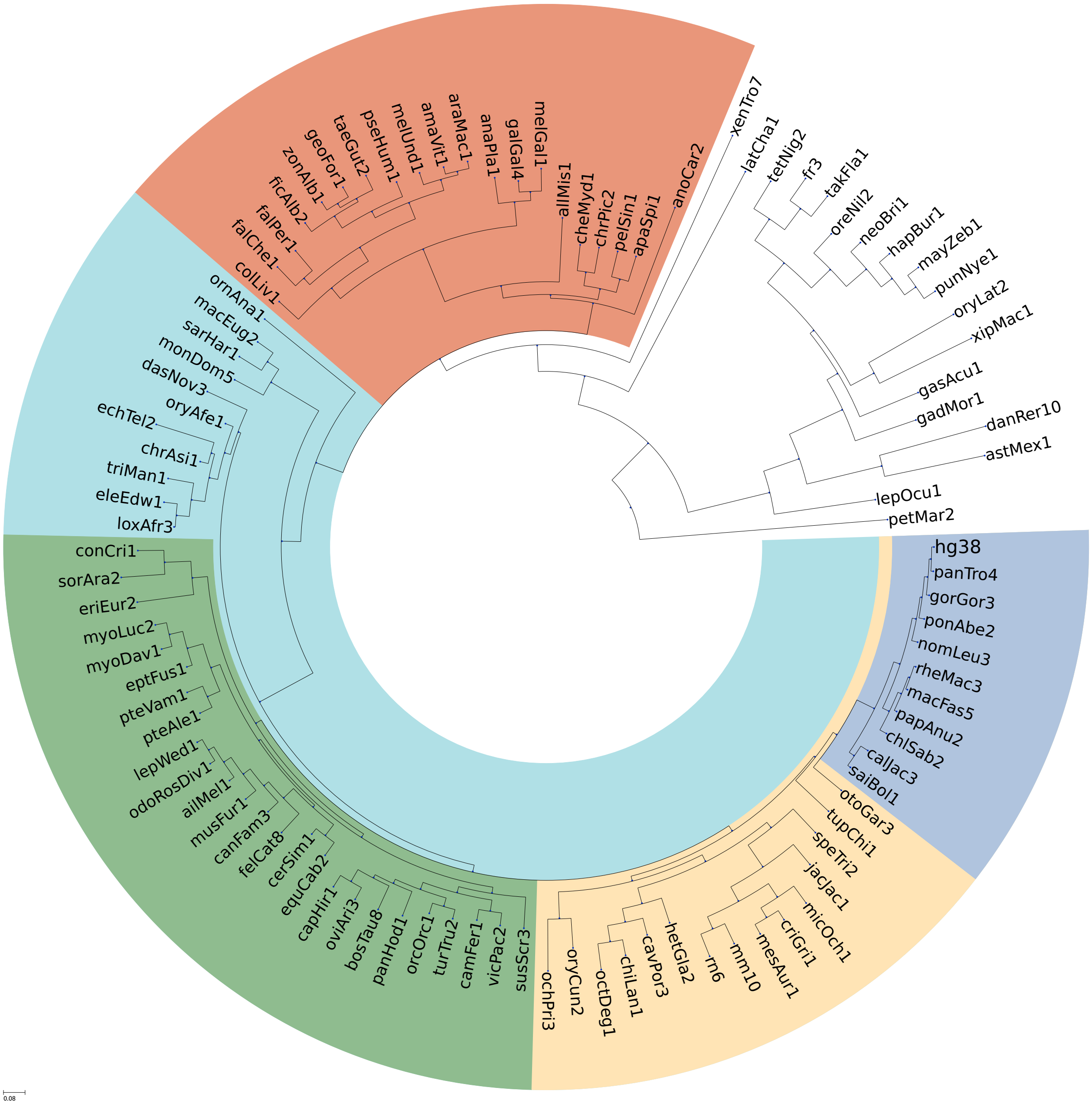

Figure S20: Phylogenetic sub divisions of species. The phylogenetic tree corresponds to a 100-way vertebrate genome alignment from the UCSC Genome Browser. Species abbreviations and genome versions are given as defined by the UCSC Genome Browser. We defined five subdivision of species based on their phylogenetic similarity and taxonomy: primates T_{11} excluding otoGar, i.e. the bush baby, (silver blue), primates and rodents T_{26} (silver blue and moccasin), closely related mammals T_{51} (silver blue, moccasin and green), mammals T_{62} (silver blue, moccasin, green and light blue) and mammals, birds and lizards T_{82} (silver blue, moccasin, green, light blue and orange).

Instances of RNA secondary structure motifs

Motif M1796580 was initially predicted as a conserved RNA secondary structure overlapping an annotated ARE. The ARE, i.e. the sequence AUUUA, is inside the loop region of structures that were predicted for the 3′-UTR of SMAD family member 2 (SMAD2) and sortilin 1 (SORT1) (Figure S23A). Both of the genes are differently expressed in gray matter and annotated as AREs. The same motif including a AUUUA sequence element in the loop region was predicted inside the 3′-UTR of microtubule associated tumor suppressor candidate 2 (MTUS2) which is also differentially expressed in gray matter. This site was annotated as structured APA in our data set, but not as an ARE.

Instances of motif M1260981 overlap with RBM47 or general RBP binding sites in eleven genes (Figure S22A) that were differentially expressed inside gray matter, the Metencephalon, the Diencephalon or the limbic lobe. These genes containing a M1260981 motif exhibit an enrichment (q-value ¡ 0.04) for nervous system development related genes (Supplementary file S7) which includes five genes, i.e. acyl-CoA synthetase long-chain family member 4 (ACSL4), N-decatylase and N-sulfotransferase 1 (NDST1), plexin A1 (PLXNA1), tetraspanin 2 (TSPAN2) and Ras associated (RalGDS/AF-6) and pleckstrin homology domains 1 (RAPH1). Four of these genes, i.e. ACSL4, PLXNA1, TSPAN2 and RAPH1, are associated with neuron projection (Supplementary file S7).

Out the 15 motifs motif M2126347 was predicted most frequently inside structured REs of differentially expressed genes in either gray matter, the Telencephalon or the Metencephalon, i.e. for 15 different genes (Figure S23C). For instance, motif M2126347 was predicted for a structured RE inside potassium voltage-gated channel subfamily H member 5 (KCNCH5) that is differentially expressed inside gray matter, the Metencephalon and the Telencephalon and in two methylcystosine dioxygenases, tet methylcystosine dioxygenase 2 (TET2) and methylcystosine dioxygenases, tet methylcystosine dioxygenase 3 (TET3) which are differentially expressed inside gray matter and the Metencephalon.

Motif M131065 was predicted in enriched RBP binding sites of four genes (Figure S22C). The instances of M1301165 predicted inside proline rich and Gla domain 1 (PRRG1), ribosomal modification protein rimK like family member B (RIMKLB) and acidic nuclear phosphoprotein 32 family member E (ANP32E) contain a UGUG sequence inside the loop region and are located in regions of either ELAV like RNA binding protein 1 (ELAVL1), CUGBP Elav-like family member 1 (CELF1) or RNA binding motif protein 47 (RBM47) binding sites. ELAVL1, CELF1 and RBM47 each comprise a three RNA recognition motif domains (RRM) [2].

Motif M054032 is one of the two motifs that do not share predicted instances with $(\mathrm{RU})_n$ repeats. Motif M054032 was predicted for structured RBP binding sites in the 3′-UTRs of SR-related CTD associated factor 1 (SCAF1), ring finger protein 138 (RNF138) and microtubule affinity regulating kinase 2 (MARK2) (Figure S21B).

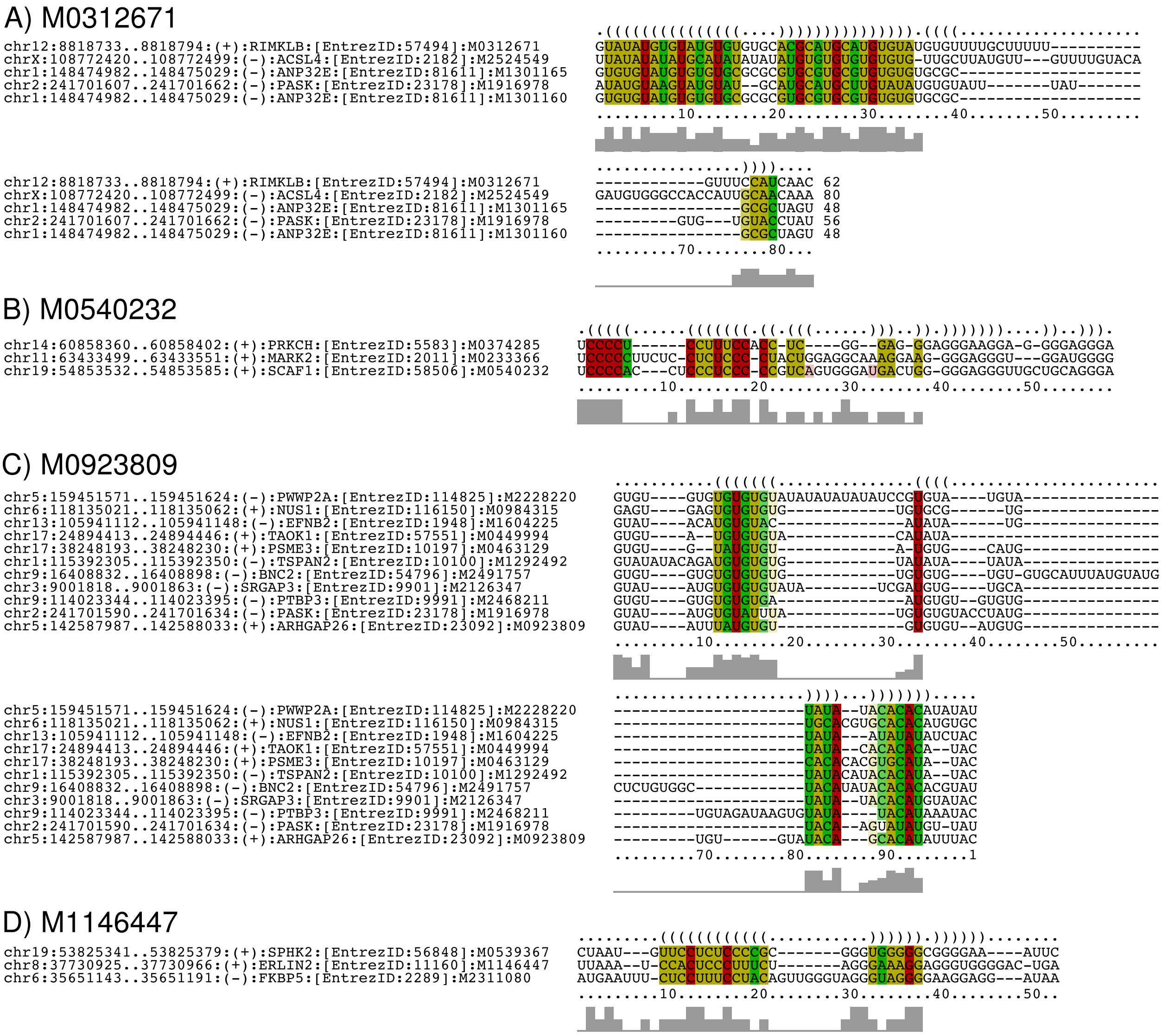

Figure S21: Predicted instances of motif M0312671, M0540323, M0923809 and M1146447. CMsearch was used to predict instances of motifs with significant E-values inside enriched structured regulatory elements of differentially expressed genes [8]. Genomic coordinates, HGNC gene symbols, Entrez Gene IDs and the motif ID is given in rows. Plots were generated with RNAalifold [1].

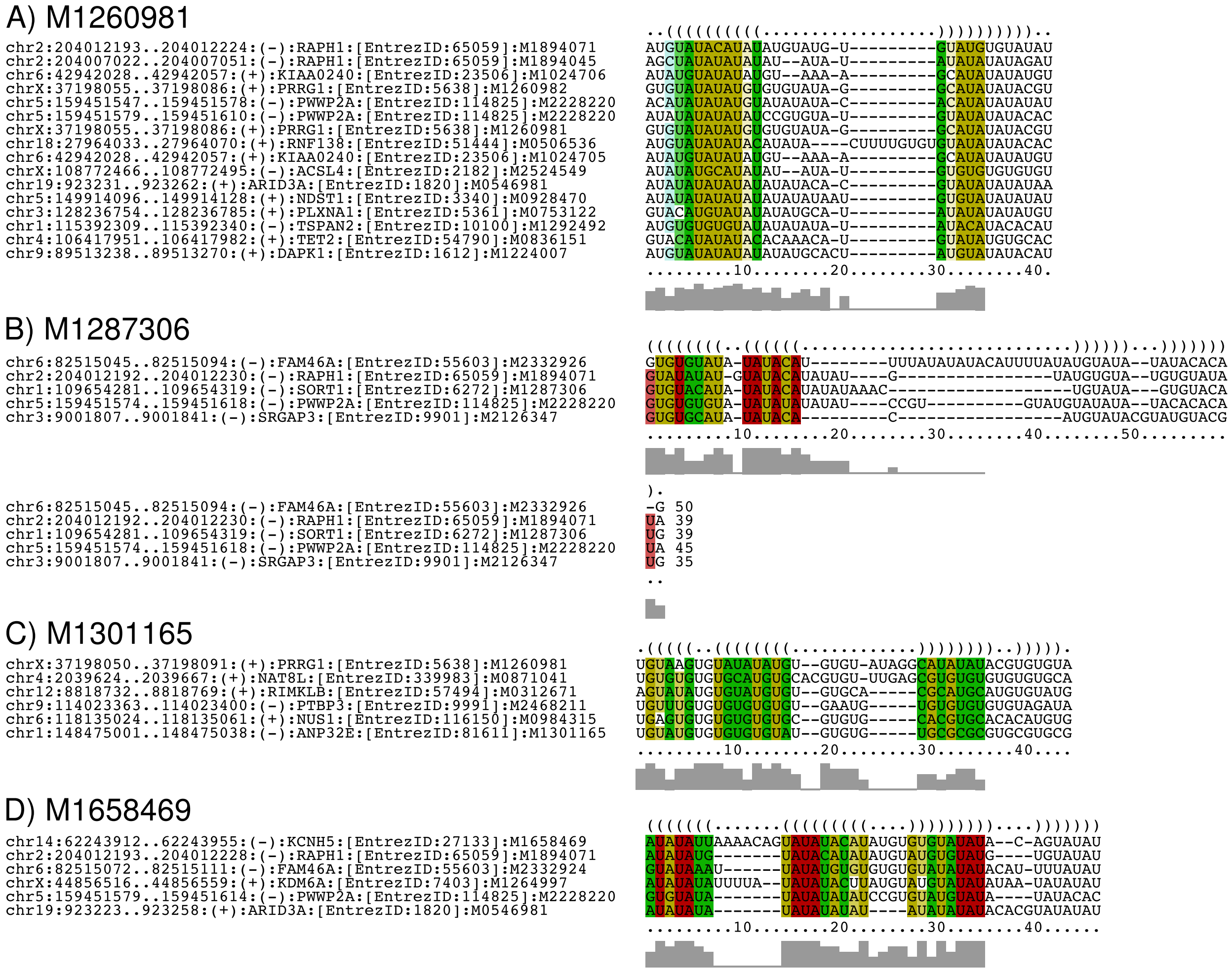

Figure S22: Predicted instances of motif M1260981, M1287306, M1301165 and M1658469. CMsearch was used to predict instances of motifs with significant E-values inside enriched structured regulatory elements of differentially expressed gens [8]. Genomic coordinates, HGNC gene symbols, Entrez Gene IDs and the motif ID is given in rows. Plots were generated with RNAalifold [1].

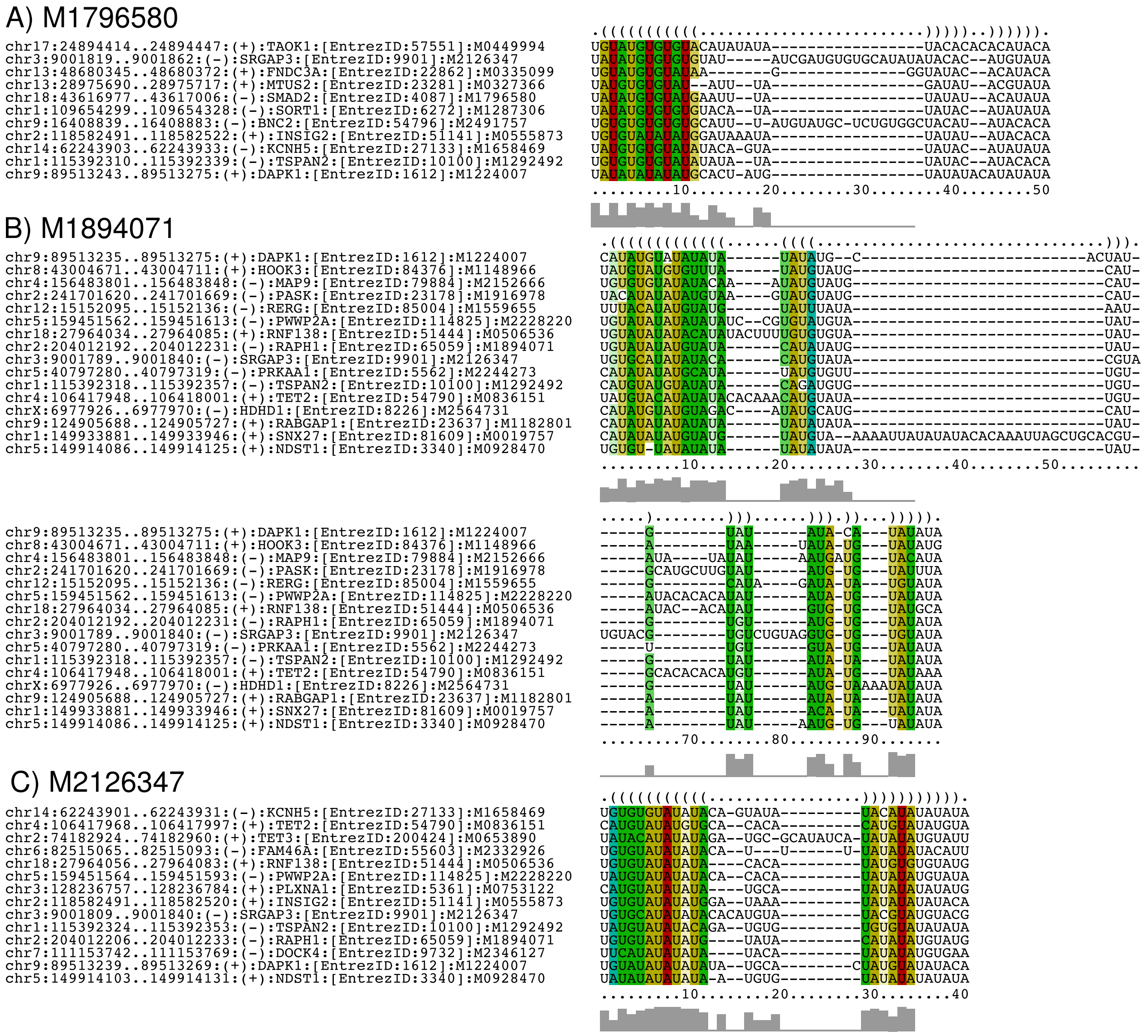

Figure S23: Predicted instances of motif M1796580, M1894071 and M2126347. CMsearch was used to predict instances of motifs with significant E-values inside enriched structured regulatory elements of differentially expressed gens [8]. Genomic coordinates, HGNC gene symbols, Entrez Gene IDs and the motif ID is given in rows. Plots were generated with RNAalifold [1].

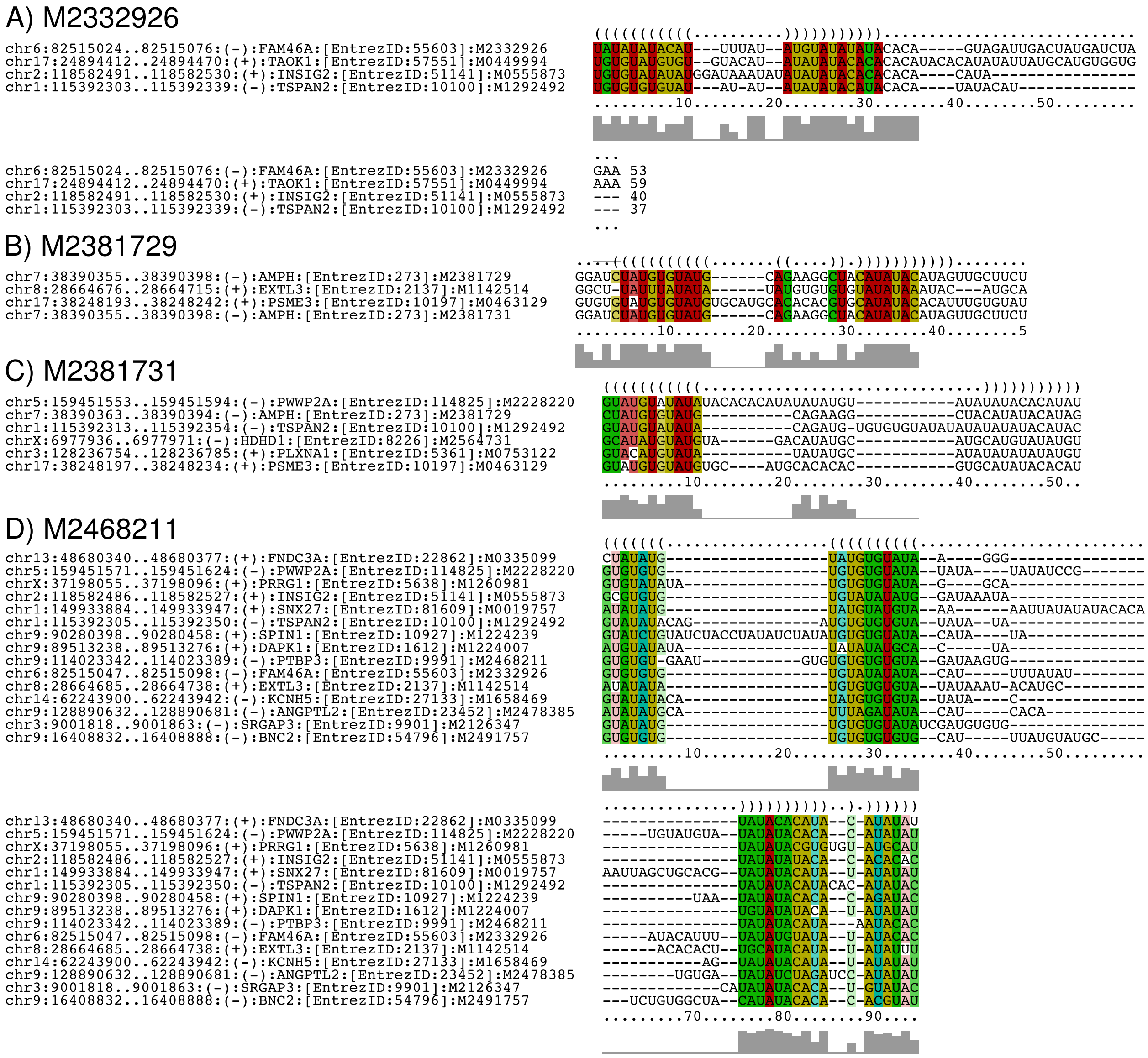

Figure S24: Predicted instances of motif M2332926, M2381729, M2381731 and M2468211. CMsearch was used to predict instances of motifs with significant E-values inside enriched structured regulatory elements of differentially expressed gens [8]. Genomic coordinates, HGNC gene symbols, Entrez Gene IDs and the motif ID is given in rows. Plots were generated with RNAalifold [1].

Extended discussion of RNA secondary structure motifs

Most instances of the $(RU)_n$-repeat motifs were predicted for enriched general RBP binding sites in differentially expressed genes. However, instances of either motif M1260981, M1894071 and M1287306 were predicted in the region of enriched RBM47 binding sites inside eight genes, i.e. ACSL4, pseudouridine 5'-phosphatase(PUDP also known as HDHD1), proline rich and Gla domain 1 (PRRG1), PWWP domain containing 2A (PWWP2A), RAPH1, ring finger protein 138 (RNF138), sorting nexin family member 27 (SNX27) and SORT1. RBM47 has been suggested to be a mediator for RNA editing [4] and to be involved

Motif ID	RE	n_{genes}	Regions	Gene symbol
M2126347	RBP	14	GM Tel MET	TSPAN2 PWWP2A DAPK1 TET3 KCNH5 NDST1 INSIG2 RNF138 PLXNA1 TET2 FAM46A RAPH1 DOCK4 SRGAP3
M1260981	RBP	11	GM LL DiE MET	TSPAN2 PWWP2A DAPK1 ACSL4 KIAA0240 NDST1 RNF138 PLXNA1 TET2 PRRG1 RAPH1
M1894071	RBP	11	GM DiE MET	TSPAN2 PWWP2A DAPK1 RABGAP1 NDST1 RNF138 TET2 RAPH1 SNX27 HDHD1 SRGAP3
M1796580	RBP	10	GM LL MET	TSPAN2 DAPK1 FNDC3A KCNH5 SMAD2 INSIG2 BNC2 TAOK1 SORT1 SRGAP3
M2468211	RBP	10	Tel DiE	TSPAN2 PWWP2A EXTL3 KCNH5 INSIG2 FAM46A PRRG1 SNX27 SRGAP3 PTBP3
M0923809	RBP	8	GM MET	TSPAN2 PSME3 PWWP2A EFNB2 ARHGAP26 BNC2 TAOK1 SRGAP3
M1260981	RBM47	5	GM LL MET	PWWP2A ACSL4 RNF138 PRRG1 RAPH1
M1287306	RBP	5	GM DiE MET	PWWP2A FAM46A SORT1 RAPH1 SRGAP3
M1894071	RBM47	5	GM MET	PWWP2A RNF138 RAPH1 SNX27 HDHD1
M2381731	RBP	5	GM Tel	TSPAN2 PSME3 PWWP2A AMPH PLXNA1
M1301165	RBP	4	GM Tel	PRRG1 RIMKLB ANP32E PTBP3
M1658469	RBP	4	GM DiE MET	PWWP2A KCNH5 FAM46A RAPH1
M2332926	RBP	4	GM Tel MET	TSPAN2 INSIG2 FAM46A TAOK1
M0312671	RBP	3	GM	ACSL4 RIMKLB ANP32E
M0540232	RBP	3	GM	MARK2 PRKCH SCAF1
M1146447	RBP	3	GM MET	ERLIN2 FKBP5 SPHK2
M1287306	RBM47	3	GM MET	PWWP2A SORT1 RAPH1
M1287306	TIA1	3	GM MET	FAM46A SORT1 RAPH1
M1796580	ARE	3	GM	SMAD2 BNC2 SORT1
M2381729	RBP	3	GM	PSME3 EXTL3 AMPH

Table S5: Regulatory elements and genes for predicted instances of motifs. RE refers to the type of regulatory element for which the structure motif was predicted including specific RBP binding sites of RBM47 and TIA1. n_{genes} is the combined number of differentially expressed genes in which the motif was predicted for the corresponding RE. Only enriched structured REs are shown in the table. Regions shows the brain regions which contain differentially expressed genes with enriched REs for which the motif was predicted. Gene symbols are HGNC gene symbols of the corresponding genes.

in mRNA stability and splicing [14]. We speculate that our results could indicate that RBM47 is involved in regulating transcripts containing the predicted $(RU)_n$-repeat motifs. Instances of motif M1287306 overlap with TIA-1 binding sites in the genes family with sequence similarity 46 member A (FAM46A), RAPH1 and SORT1. In the loop region of the structure prediction inside FAM46A, we observed two AUUUUA sequences which are very similar to an AU-rich element, AUUUA sequence (Figure S22B, plots were generated with RNAalifold [1]). Binding of TIA1 to an AU-rich element has been implicated in the down-regulation of the expression of tumor necrosis factor α (TNFA) [10]. Note that we did not find similar sequences inside the predicted instances of motif M1287306 in RAPH1 and SORT1, but this finding may suggest that TIA1 also binds to AU-element like sequences or AU-rich sequences in general. We observed that instances of motif M1301165 which contain a UGUG sequence in the loop region overlap with RBPs that contain RRM domains. This may suggest semi-specific binding for the M1301165 motif. Further experiments are necessary for verifying or negating these hypotheses.

References

[1] S. H. Bernhart, I. L. Hofacker, S. Will, A. R. Gruber, and P. F. Stadler. RNAalifold: improved consensus structure prediction for RNA alignments. *BMC bioinformatics*, 9:474, Nov 2008.

[2] K. B. Cook, H. Kazan, K. Zuberi, Q. Morris, and T. R. Hughes. Rbpdb: a database of rna-binding specificities. *Nucleic acids research*, 39:D301–D308, Jan 2011.

[3] A. Di Fonzo, M. Dekker, P. Montagna, A. Baruzzi, E. Yonova, L. C. Guedes, A. Szczerbinska, T. Zhao, L. Dubbel-Hulsman, C. Wouters, et al. FBXO7 mutations cause autosomal recessive, early-onset parkinsonian-pyramidal syndrome. *Neurology*, 72(3):240–245, 2009.

[4] N. Fossat, K. Tourle, T. Radziewic, K. Barratt, D. Liebhold, J. B. Studdert, M. Power, V. Jones, D. A. F. Loebel, and P. P. L. Tam. C to u rna editing mediated by apobec1 requires rna-binding protein rbm47. *EMBO reports*, 15:903–910, Aug 2014.

[5] Y. Gelfand and M. G. Kaplitt. Gene therapy for psychiatric disorders. *World neurosurgery*, 80(3):S32–e11, 2013.

[6] B. Gong, F. Chen, Y. Pan, I. Arrieta-Cruz, Y. Yoshida, V. Haroutunian, and G. M. Pasinetti. SCFFbx2-E3-ligase-mediated degradation of BACE1 attenuates Alzheimer's disease amyloidosis and improves synaptic function. *Aging cell*, 9(6):1018–1031, 2010.

[7] B. Knoell and A. Nordheim. Functional versatility of transcription factors in the nervous system: the SRF paradigm. *Trends Neurosci*, 32(8):432–442, Aug 2009.

[8] E. P. Nawrocki and S. R. Eddy. Infernal 1.1: 100-fold faster RNA homology searches. *Bioinformatics*, 29(22):2933–2935, Nov 2013.

[9] D. E. Nelson, S. J. Randle, and H. Laman. Beyond ubiquitination: the atypical functions of Fbxo7 and other F-box proteins. *Open biology*, 3(10):130131, 2013.

[10] M. Piecyk, S. Wax, A. R. Beck, N. Kedersha, M. Gupta, B. Maritim, S. Chen, C. Gueydan, V. Kruys, M. Streuli, and P. Anderson. Tia-1 is a translational silencer that selectively regulates the expression of tnf-alpha. *The EMBO journal*, 19:4154–4163, Aug 2000.

[11] A. Schroeder, O. Mueller, S. Stocker, R. Salowsky, M. Leiber, M. Gassmann, S. Lightfoot, W. Menzel, M. Granzow, and T. Ragg. The RIN: an RNA integrity number for assigning integrity values to RNA measurements. *BMC Mol Biol*, 7:3, 2006.

[12] C. Stritt, S. Stern, K. Harting, T. Manke, D. Sinske, H. Schwarz, M. Vingron, A. Nordheim, and B. Knöll. Paracrine control of oligodendrocyte differentiation by SRF-directed neuronal gene expression. *Nat Neurosci*, 12(4):418–427, Apr 2009.

[13] S. M. Sunkin, L. Ng, C. Lau, T. Dolbeare, T. L. Gilbert, C. L. Thompson, M. Hawrylycz, and C. Dang. Allen Brain Atlas: an integrated spatio-temporal portal for exploring the central nervous system. *Nucleic Acids Res*, 41(Database issue):D996–D1008, Jan 2013.

[14] S. Vanharanta, C. B. Marney, W. Shu, M. Valiente, Y. Zou, A. Mele, R. B. Darnell, and J. Massagué. Loss of the multifunctional RNA-binding protein RBM47 as a source of selectable metastatic traits in breast cancer. *Elife*, 3, 2014.
